# D-Xylose Sensing in *Saccharomyces cerevisiae*: Insights from D-Glucose Signaling and Native D-Xylose Utilizers

**DOI:** 10.3390/ijms222212410

**Published:** 2021-11-17

**Authors:** Daniel P. Brink, Celina Borgström, Viktor C. Persson, Karen Ofuji Osiro, Marie F. Gorwa-Grauslund

**Affiliations:** 1 Applied Microbiology, Department of Chemistry, Lund University, P.O. Box 124, SE-221 00 Lund, Sweden; celina.tufvegren@utoronto.ca (C.B.); Viktor.Persson@tmb.lth.se (V.C.P.); karenosiro@gmail.com (K.O.O.); 2 BioZone Centre for Applied Bioscience and Bioengineering, Department of Chemical Engineering and Applied Chemistry, University of Toronto, 200 College St., Toronto, ON M5S 3E5, Canada; 3 Genetics and Biotechnology Laboratory, Embrapa Agroenergy, Brasília 70770-901, DF, Brazil

**Keywords:** *Saccharomyces cerevisiae*, d-xylose, sugar sensing, sugar signaling, non-native substrate, signaling network engineering, synthetic signaling circuits

## Abstract

Extension of the substrate range is among one of the metabolic engineering goals for microorganisms used in biotechnological processes because it enables the use of a wide range of raw materials as substrates. One of the most prominent examples is the engineering of baker’s yeast *Saccharomyces cerevisiae* for the utilization of d-xylose, a five-carbon sugar found in high abundance in lignocellulosic biomass and a key substrate to achieve good process economy in chemical production from renewable and non-edible plant feedstocks. Despite many excellent engineering strategies that have allowed recombinant *S. cerevisiae* to ferment d-xylose to ethanol at high yields, the consumption rate of d-xylose is still significantly lower than that of its preferred sugar d-glucose. In mixed d-glucose/d-xylose cultivations, d-xylose is only utilized after d-glucose depletion, which leads to prolonged process times and added costs. Due to this limitation, the response on d-xylose in the native sugar signaling pathways has emerged as a promising next-level engineering target. Here we review the current status of the knowledge of the response of *S. cerevisiae* signaling pathways to d-xylose. To do this, we first summarize the response of the native sensing and signaling pathways in *S. cerevisiae* to d-glucose (the preferred sugar of the yeast). Using the d-glucose case as a point of reference, we then proceed to discuss the known signaling response to d-xylose in *S. cerevisiae* and current attempts of improving the response by signaling engineering using native targets and synthetic (non-native) regulatory circuits.

## 1. Introduction

Metabolic engineering has enabled various microorganisms to grow on and convert non-native carbon sources into useful bulk and fine chemicals through recombinant expression of heterologous pathways identified in other species. A remarkable amount of successful engineering strategies that enable yeast and bacteria to grow on substrates they cannot naturally assimilate have been published in the last decades and have broadened the possibilities of applied biotechnology (with some examples including [1,2,3,4]; see also reviews by [5,6,7]). However, while many new proof-of-concept studies emerge every year and increase the diversity of available cell factories, improving and optimizing these systems to reach industrially and societally relevant levels of production has proven a much more difficult challenge [8,9,10,11].

One of the most prominent examples concerns the engineering of baker’s yeast *Saccharomyces cerevisiae* to ferment the five-carbon (pentose) sugar d-xylose (representing up to 25% of the sugars present in the renewable feedstock lignocellulose [12]), a flagship project of metabolic engineering dating back to the end of the 1970s [13]. The first proof-of-concept study that could demonstrate d-xylose utilization was published in 1990 [14] and it has since been followed by substantial research efforts to increase the d-xylose utilization and d-glucose/d-xylose co-utilization rates to levels required for the implementation of economically feasible industrial processes.

**Figure 1:**
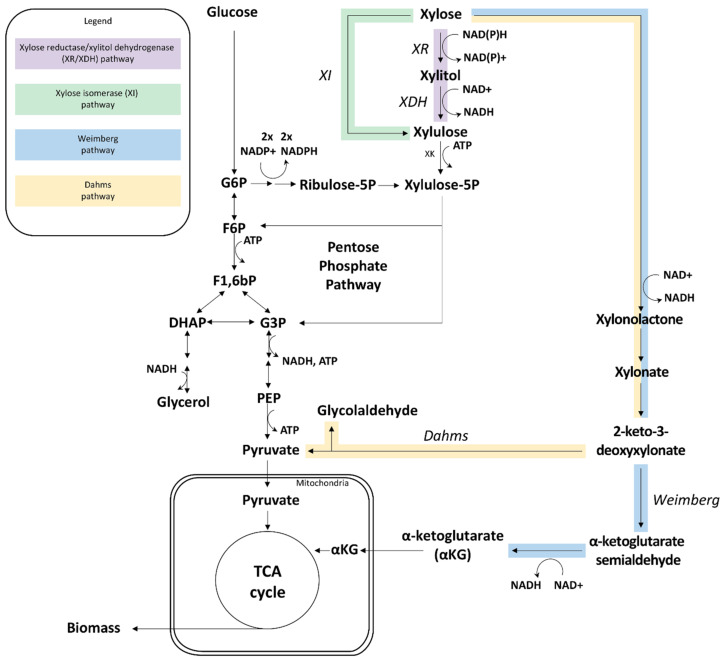
Overview of the four heterologous d-xylose pathways that have been introduced in *S. cerevisiae* to date, and their connections to glycolysis and the TCA cycle. G6P: glucose-6-phosphate; F6P: fructose-6-phosphate; F1,6bP: fructose-1,6-bisphosphate; DHAP: dihydroxyacetone phosphate; G3P: glyceraldehyde 3-phosphate; PEP: phosphoenolpyruvate; XK: Xylulokinase; TCA cycle: tricarboxylic acid cycle.

Four types of exogenous metabolic pathways have been implemented in *S. cerevisiae* (Figure 1): the oxidoreductive xylose reductase/xylitol dehydrogenase (XR/XDH) pathway, the xylose isomerase (XI) pathway, the oxidative Dahms pathway and the oxidative Weimberg pathway [14,15,16,17], each presenting different engineering challenges. In addition to the pathway itself, several key modifications were found to be essential for efficient d-xylose utilization, notably the upregulation of xylulokinase and the non-oxidative pentose phosphate pathway genes for the XR/XDH and XI strategies, the balancing of cofactor usage between XR and XDH in the XR/XDH pathway, the increase in XI activity by the identification of better XIs and by increasing *xylA* gene (encoding XI) copy numbers, or the implementation of an alternative dehydrogenase in the Weimberg pathway [13,17,18,19,20,21]. However, in the case of ethanol production from d-xylose, the best reported XR-XDH and XI strains are still far behind in terms of d-xylose consumption rate and specific ethanol productivity as compared to the d-glucose data (Table 1). The lack of dedicated d-xylose membrane transporters has also long been considered as a main bottleneck in *S. cerevisiae* d-xylose utilization [22,23,24], especially for d-glucose/d-xylose co-consumption because d-xylose is transported via the same hexose transporter as d-glucose and thereby competes with d-glucose for transport. This has been supported by studies showing that the specific d-xylose consumption rate increased with increased extracellular d-xylose concentration [25,26] as well as when expressing heterologous transporters [27]. Significant advances are currently being made in the identification and engineering of novel d-xylose transporters with improved kinetics and substrate specificities (recently comprehensively reviewed by Nijland and Driessen [28], with new studies continuing to be published at a high rate [29,30,31,32,33]). However, *S. cerevisiae* XR/XDH and XI strains still ferment d-xylose at a fraction of the rate of d-glucose and d-xylose is still consumed after d-glucose depletion in d-glucose/d-xylose co-cultivations, highlighting other challenges for d-xylose utilization.

An increasing number of studies have pointed to the unusual physiological response to d-xylose in the xylose-engineered yeast strains: the cells ferment ethanol from d-xylose but exhibit a respiratory response while doing so [34,35,36,37,38]. This has led to the hypothesis that *S. cerevisiae* may not recognize this foreign pentose sugar as a fermentable sugar [37]. Consequently, the sugar sensing, signaling and regulation systems of the yeast may need to be adjusted to respond to d-xylose [39]. In the present review we summarize the current knowledge on the effect of d-xylose on the sugar signaling networks. We first describe sugar sensing in *S. cerevisiae* and establish the baseline case of how the yeast senses its preferred sugar, d-glucose (Section 3). Then, the currently known effects of d-xylose on the sugar signaling networks in wild-type and XR/XDH- or XI-engineered yeast strains are summarized (Section 4), with special emphasis on how they differ from the d-glucose response. The *S. cerevisiae* d-xylose signaling response is then further contrasted by summarizing the current knowledge of d-xylose sensing in a few other microbes capable of naturally utilizing d- xylose (Section 4.2). Finally, the current and future states of d-xylose signaling engineering are discussed from three different but complementary perspectives: engineering the native signaling network, constructing synthetic signaling circuits, and computational modeling of sugar signaling (Section 5).

## 2. What Is Sugar Sensing and Signaling?

### 2.1. Signaling Networks Control Cellular Functions in Response to Environmental Changes

**Figure 2:**
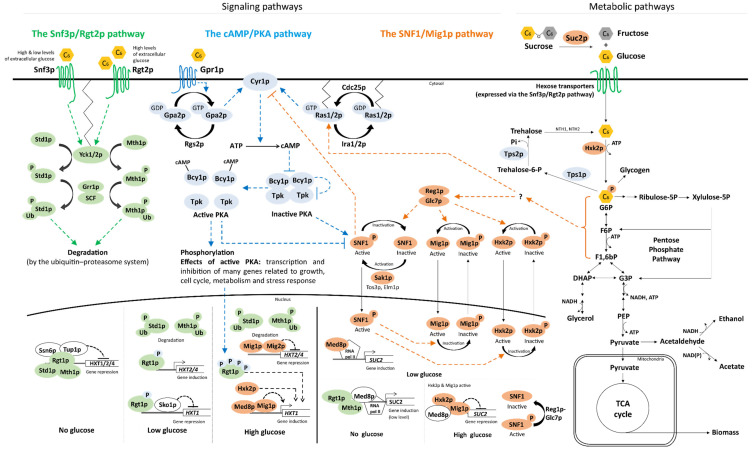
Overview of the key signal transduction events in three major sugar signaling pathways (Snf3p/Rgt2p, cAMP/PKA, SNF1/Mig1p) in *S. cerevisiae* and their connection to d-glucose and d-xylose metabolism. The XR/XDH and XI pathways are also shown in detail, whereas only the major connections are shown for the Weimberg and Dahms pathways. Three different carbon source availability cases are depicted: no d-glucose, low d-glucose and high d-glucose concentrations. Colored shapes: proteins under the control of the signaling pathway of the same color. Arrows with arrowheads: induction; arrows with hammerheads: repression; dashed arrows: signaling; solid arrows: metabolic or transport reactions; circles with P: phosphorylation (the color of the circle indicates which signaling pathway controls the phosphorylation); circles with Ub: ubiquitination; yellow hexagons: d-glucose; grey hexagons: d-fructose. Green shapes: Snf3p/Rgt2p pathway; blue shapes: cAMP/PKA pathway; orange shapes: SNF1/Mig1p pathway; white shapes (Ssn6p-Tup1p, Sko1p, Med8p): transcription factors involved with basal transcription machinery, high osmolarity/glycerol (HOG) pathway and Hxk2p-activated gene regulation, respectively. Zig-zag lines attached to Yck1/2p and Ras1/2p indicate membrane anchoring. See text in Section 3 for more details. Adapted from [77,78,79].

The purpose of signaling pathways is to sense environmental stimuli and transmit signals to intracellular targets that in turn regulate the cellular response [46]. Key signaling pathways regulate a wide number of cellular functions in *S. cerevisiae* [47], such as sensing of nutrients (e.g., sugars, nitrogen, phosphate [48]), stress response [49,50,51,52,53], growth [48,54] or mating [55]. Unlike metabolic pathways that consists of enzymatic reactions where substrates are converted to products, signaling pathways consists of signal transduction cascades controlled by sensors, transducers and actuators [56]. Signal cascades (Figure 2) can be divided in three parts: (i) signaling molecules binding to receptors (in many cases transmembrane proteins that sense extracellular molecules) and initiation of the cascade; (ii) signal transduction driven by interactions between the proteins within the pathway (using mechanisms such as phosphorylation, ubiquitination, cellular translocation [57,58,59,60]), and small signal carrying molecules such as cyclic AMP (cAMP) and ions such as sodium, magnesium and calcium [61,62]; and (iii) induction and repression of genes, or activation and inactivation of proteins and enzymes (the end-outcome of the signal cascade). In addition to their specific functions, many signaling networks interact in a phenomenon known as cross-talk (signal transduction between signaling pathways [56]). Signaling networks can also elicit heterogeneous responses across a cell population [56,63,64].

### 2.2. Main Pathways Involved in S. cerevisiae Sugar Sensing and Signaling

To cope with varying carbon source availability, *S. cerevisiae* has evolved the capacity to metabolize a wide range of mono- and disaccharides, such as d-glucose, d-galactose, d-fructose, d-mannose, sucrose and maltose [65,66] and has consequently developed complex signaling systems to respond to and prioritize between these sugars. d-Glucose is the preferred sugar for *S. cerevisiae* and, as such, it has a strong regulatory effect on many cellular processes [48,67,68]. The preference for d-glucose over other carbon sources is manifested by how its presence leads to the inactivation of the metabolic pathways involved in the assimilation of other carbon sources; this phenomenon is known as carbon catabolite repression (CCR), or in this specific case glucose (catabolite) repression [69]. This effectively means that in any cultivation medium containing d-glucose and another utilizable sugar, *S. cerevisiae* will not start catabolizing the co-sugar until d-glucose has been depleted or is close to depletion [69,70,71,72,73,74,75]. Glucose repression is a signal to induce genes and activate enzymes of the glycolysis and simultaneously inactivate enzymes/repress genes in the gluconeogenesis and the pathways for utilization of alternative carbon sources; likewise, the opposite signal (inactivation/repression of glycolytic enzymes/genes and activation/induction of gluconeogenesis and enzymes/genes involved in alternative carbon source utilization) is transduced upon d-glucose depletion [69,76].

Being the preferred sugar that triggers CCR, d-glucose is logically highly involved in the sugar signaling networks and d-glucose sensing is the subject of many reviews [66,67,68,80,81,82,83,84]. Although mechanistic details are refined every year, the current model of d-glucose sensing is very mature, with three main sugar signaling networks identified in baker’s yeast—and further detailed in Section 3 below and in Figure 2: the Snf3p/Rgt2p pathway that senses extracellular d-glucose and responds by inducing expression of hexose transporters that in turn transport d-glucose inside the cell; the SNF1/Mig1p pathway that is activated in the absence of d-glucose and regulates genes related to alternative (non-glucose) sugar utilization; and the cAMP/PKA pathway that regulates growth, cell cycle, metabolism and stress response [67]. Other *S. cerevisiae* signaling pathways are also partly involved in sugar sensing: (i) the high osmolarity/glycerol (HOG) pathway, which is one of the four mitogen-activated protein kinase (MAPK) pathways, responds to osmotic stress such as high environmental concentrations of salts and sugars [51,85]; (ii) the filamentous growth pathway (also part of MAPK) that triggers pseudohyphal growth upon nutrient starvation to scavenge nutrients, is activated via one of the constituents of the cAMP/PKA pathway (Ras2p) [51,86]; (iii) the target of rapamycin (TOR) pathway that senses nitrogen availability and co-operates with the d-glucose sensing of the cAMP/PKA pathway to regulate, e.g., cell growth [48,87]; and (iv) the d-galactose (*GAL)* regulon that allows for expression of genes needed for d-galactose catabolism when CCR is relieved [88,89].

## 3. What Happens on d-Glucose, the Model Case for Sugar Signaling?

To be able to discuss the current knowledge on the d-xylose signaling response in *S. cerevisiae* (Section 4), we first need to establish the mechanistic details of the signaling cascades triggered in response to varying availability of d-glucose, the model case for *S. cerevisiae* sugar signaling. Sensing of different d-glucose levels via the sugar signaling pathways results in two major levels of regulation: induction and repression of target genes, as well as activation and inactivation of enzymes and other proteins. The transcriptional regulation typically occurs at the end of a signal cascade, where the signal reaches regulatory proteins known as transcription factors (TFs). These proteins bind to DNA and induce or repress transcription by interactions with RNA polymerase II and histones; additional proteins called co-regulators also interact with TFs and are also involved in this process [90]. The regulation of enzymes and proteins in these pathways mainly occur through phosphorylation, either as mechanism of signal transduction (e.g., in the SNF1/Mig1p pathway [91]), or as a means of control over other cellular pathways (as for instance in the case of the cAMP/PKA pathway [92]). Ubiquitinations are also used to regulate protein activity by marking them for degradation (e.g., in the Snf3p/Rgt2p pathway [93]). Whereas all three major sugar sensing pathways may be affected by intracellular events occurring during catabolism, extracellular d-glucose is sensed directly by two pathways: Snf3p/Rgt2p and cAMP/PKA [94,95]. The literature has generally focused on investigating three general cases with separate signaling outcomes: high d-glucose concentrations, low d-glucose concentrations, and absence of d-glucose (Figure 2). Different studies have however used slightly different concentrations of d-glucose for the different conditions, so for the sake of this review, we defined the different ranges as 10–20 g L^−1^, 1–5 g L^−1^, and 0 g L^−1^, respectively. Below, we first review the effect of d-glucose on each signaling pathway before summarizing cross-talk and system-wide effects of d-glucose sensing in Section 3.5.

### 3.1. d-Glucose Sensing by the Snf3p/Rgt2p Pathway Regulates Hexose Transporter Gene Expression

The Snf3p/Rgt2p pathway responds to varying levels of extracellular d-glucose using the transmembrane d-glucose sensors Snf3p and Rgt2p (Figure 2), eventually leading to the regulation of the expression of hexose transporter genes [96,97]. The Rgt2p and Snf3p receptors have different affinities for d-glucose and together cover the sensing of a span of extracellular d-glucose concentrations: the Rgt2p sensor is activated by high concentration of d-glucose (e.g., 40 g L^−1^ in [94]), which triggers a signaling cascade resulting in the expression of genes encoding hexose transporters with low affinity to d-glucose (such as *HXT1*). The Snf3p sensor covers a wider spectrum, as it responds to both high and low d-glucose concentrations (e.g., 1 and 40 g L^−1^ in [94]). Snf3p activation results in the transcription of genes for hexose transporters with high affinity to d-glucose, e.g., *HXT2/4* [94,98]. The third case, absence of d-glucose, results in repression of genes for both high and low affinity hexose transporters [97], which leaves room for expression of transporters of other sugars (e.g., d-galactose [88]).

It has been suggested that the Snf3p and Rgt2p sensors evolved from hexose transporters that have lost their capacity to transport sugars [98]; indeed, attaching the tail of Snf3p to either Hxt1p or Hxt2p transforms the transporters into d-glucose signaling entities [99]. It has furthermore been hypothesized that Snf3p and Rgt2p might sense the ratio of internal and external concentrations of d-glucose [100]. In practice, binding of d-glucose to the Snf3p and Rgt2p transmembrane receptors leads to a conformational change in their respective C-terminal cytosolic tails; a 17 amino acid conserved repeat in the tail is thought to confer signaling strength as it is found once in the Rgt2p (which only senses high d-glucose concentrations) and twice in Snf3p (which senses both high and low d-glucose levels) [94]. The d-glucose induced conformational changes transduce an activation signal to the membrane-bound Yck1p/2p casein kinases which phosphorylate the Mth1p and Std1p transcriptional repressors [93,96,101]. The two proteins form a repressor complex together with Rgt1p and co-repressors Tup1p and Ssn6p that regulates the expression of several hexose transporters [94,102]. Their phosphorylation sends a signal for ubiquitination to the SCF^Grr1^ (Skp1p, Cdc53p/Cul1p-Grr1p [103]) ubiquitin ligase complex, which leads to their subsequent degradation in the proteasome (Figure 2) [93,104]. Another layer of signal complexity is achieved by the bi-functionality of Rgt1p: the protein only acts as a transcriptional repressor in the absence of d-glucose and is converted to a transcriptional inducer by phosphorylation (e.g., by PKA) once d-glucose is present (Figure 2) [94,105,106].

Many sugar transporters (e.g., *HXT1/2/4/6*) are induced upon d-glucose sensing by the Snf3p/Rgt2p pathway [96,107,108], and deletion of *SNF3*/*RGT2* results in growth defects during high d-glucose conditions [99,109], likely due to insufficient expression of *HXT* genes. However, a few transporters are subject to alternative transcriptional regulation: (i) the *GAL2* galactose transporter gene, whose product is capable of transporting d-glucose and d-xylose, is instead regulated by the galactose regulon [88,110,111], (ii) *HXT3* is induced by d-glucose regardless of its concentration [107], and (iii) *HXT5* is regulated by growth rates [112]. The Snf3p/Rgt2p pathway also regulates expression of the transcriptional repressor genes *MIG2/3* [113,114]. Mig2p/3p bind to similar DNA motifs as their paralog Mig1p (a key element of the SNF1/Mig1p pathway, discussed in Section 3.2 below), and regulate expression of, e.g., *HXT2/4, MTH1* and *MIG1* [114]. However, Mig1p and Mig2p/3p regulate different targets and are under control of different pathways (SNF1/Mig1p and Snf3p/Rgt2p, respectively) and thus cover slightly different d-glucose signals.

### 3.2. The SNF1/Mig1p Pathway Represses Transcription of Genes Related to Alternative Carbon Sources upon Sensing of d-Glucose

The SNF1/Mig1p signaling pathway is the main regulator of d-glucose CCR, tightly repressing genes involved in growth on alternative carbon sources such as sucrose, d-galactose, glycerol, and ethanol, when d-glucose is available.

SNF1/Mig1p signals through a kinetic equilibrium between a phosphorylated and a dephosphorylated state. The pathway responds to high d-glucose concentrations by shifting this equilibrium towards dephosphorylation of the pathway constituents Hxk2p, Mig1p, Snf1p and Reg1p by the phosphatase Glc7p. d-Glucose depletion, however, leads to phosphorylation of the same targets by the Snf1p and Sak1p kinases. The exact mechanism behind this shifting kinetic equilibrium has yet to be fully elucidated, but it has been shown that the response to d-glucose is dependent on the formation of glucose-6-phosphate through the first step of glycolysis. This reaction is catalyzed by hexo- and glucokinases, with hexokinase 2 (Hxk2p) being the main contributor at high d-glucose levels [115]. In addition to this, Hxk2p also plays an important role as a signaling protein in the SNF1/Mig1p pathway and a regulator of gene expression [116]. Ma and colleagues screened a library of 24 different Hxk2p variants and could correlate the degree of d-glucose repression with the residual phosphorylating activity of d-glucose [117].

Phosphorylation/dephosphorylation as a mechanism for signal transduction is able to affect target proteins in widely varied ways, leading, for example, to enzymatic activation or deactivation, protein degradation, subcellular shuttling or modifications in protein-protein or protein-DNA interactions. This renders the SNF1/Mig1p pathway much more dynamic in its response to changes to d-glucose levels than the other two sugar signaling pathways. Specific effects of SNF1/Mig1p signaling on its major targets and constituents are discussed below. d-Glucose-induced dephosphorylation of Hxk2p activates the regulatory function of the protein, as a subpopulation of the protein migrates to the nucleus where it acts as a co-regulator interacting with Med8p, Rgt1p and the dephosphorylated Mig1p to repress genes involved in alternative carbon source utilization, such as *SUC2* and the *GAL* genes (Figure 2) [118]. The heterotrimeric protein complex SNF1 (consisting of the Snf1p α-unit, the Snf4p γ-unit and any of the following β-units: Sip1p, Sip2p, or Gal83p) functions to fine-tune the d-glucose signal to Hxk2p and Mig1p [119,120]. d-Glucose-induced dephosphorylation of the Snf1p subunit triggers interaction between its kinase- and regulatory domains, which destabilizes and inactivates the kinase function of the complex [121,122]. The kinase targets of SNF1 include Hxk2p, Mig1p and Reg1p, and the d-glucose induced inactivation of this kinase leads to a further tightened CCR when d-glucose is present at high levels. The phosphorylation state of Snf1p, together with the type of the β-unit, also affects the subcellular localization of the complex; the complex is found in the cytosol at high d-glucose levels but, under low d-glucose levels, SNF1 complexes containing Gal83p, Sip1p and Sip2p are localized in the nucleus, vacuoles and cytosol, respectively [123]. As Reg1p regulates Glc7p phosphatase activity and specificity, the dephosphorylation of Reg1p by Glc7p further weakens interaction with the phosphatase, increasing its activity in a positive feedback loop [124].

Sensing of d-glucose depletion through the SNF1/Mig1p pathway leads to decreased Glc7p activity while the Sak1p kinase is activated, phosphorylating Snf1p. Active SNF1 complex, in turn, phosphorylates Hxk2p and Mig1p, reversing their nucleocytoplasmic shuttling and alleviating repression of target genes [125]. SNF1 also controls the activation state of a number of genetic activators. Notably, when d-glucose is depleted, SNF1 phosphorylates and activates Adr1p, Cat8p and Sip4 initiating transcription of gluconeogenetic genes [126].

Attempts to disturb CCR have been made by deleting genes from some of the key regulators discussed above. The deletion of *GLC7* was found to be lethal, possibly due to G_2_/M cell cycle arrest [127]. Deletion of the *HXK2* gene, however, only led to loss of d-glucose repression for genes involved in sucrose and d-galactose assimilation; in this strain, growth on d-glucose was maintained thanks to the presence of Hxk1p and Glk1p, two homologs lacking the regulatory function [116]. A similar pattern was observed when deleting genes from the SNF1 complex subunits (such as *SNF4, SIP1*, *SIP2* and *GAL83*), which resulted in viable strains but with a modified d-glucose repression pattern [128].

### 3.3. The cAMP/PKA Pathway Triggers a Phosphorylation Cascade after Sensing d-Glucose

The cAMP/PKA pathway senses extracellular d-glucose through the transmembrane receptor Gpr1p and intracellular d-glucose-derived signals via Ras1p/2p [48,129]. The signals from the Gpr1p and Ras1p/2p branches converge on adenylate cyclase (Cyr1p) that catalyzes the formation of cAMP (Figure 2). The transition from absence to presence of d-glucose triggers a transient pulse of cAMP that activates the protein kinase A (PKA) protein complex [68,130], that subsequently regulates several cellular functions, including growth, metabolism, stress response and cellular homeostasis (summarized in Table 2) by phosphorylation of enzymes and TFs [48].

Gpr1p can sense extracellular d-glucose and, to a lower degree, sucrose, but it does not respond to d-galactose and d-fructose; and is inhibited by d-mannose [95]. Gpr1p transmits its signals to the G-protein Gpa2p, which triggers the replacement of a Gpa2p-bound GDP with a GTP [149,150]. GTP-bound, activated Gpa2p transduces the d-glucose-induced signal to Cyr1p (Figure 2) [98,151]. The Ras1p/2p branch of the pathway functions in a similar manner but senses intracellular signals: the Ras1p/2p G-proteins also bind GTP upon activation, which allows for interaction with Cyr1p and initiation of a cAMP formation cascade [98]. In the case of Ras1p/2p, the bound GTP has been shown to be an essential part of the signal transduction to Cyr1p [152]. The exact mechanisms of how the Ras1p/2p branch senses intracellular d-glucose-derived signals are not fully understood, but intracellular acidification has been shown to shift the Ras1p/2p GTP:GDP ratio towards increased Ras1p/2p activation and subsequent cAMP pulses [153]. The immediate phosphorylation of d-glucose to glucose-6-phosphate by hexokinases upon transport inside the cell may explain this phenomenon since glucose-6-phosphate is a weak acid (pK_a_ = 1.4) that might cause temporary drops in intracellular pH. Other glycolytic intermediates have been reported to have an effect on the cAMP/PKA pathway and are discussed below in Section 3.6. Ras1p/2p also seems to change its cellular localization depending on d-glucose availability, as a Ras-GFP fusion biosensor was found in the plasma membrane and nucleus in the presence of d-glucose, but in the mitochondrion in its absence [154]. In addition to the different types of signals sensed by the Gpr1p and Ras1p/2p branches, the two branches also respond to different d-glucose concentrations: the Gpr1p senses higher concentrations above 4–20 g L^−1^ while Ras1p/2p is responsive to sugar levels equivalent to 0.2–2 g L^−1^ extracellular d-glucose [130,153,155,156].

The function of the cAMP pulse is to convert the inactive PKA complex to its active form (Figure 2). Inactive PKA consists of two catalytical subunits (combinations of Tpk1p/2p/3p) that are being inhibited by two Bcy1p subunits [157]. As cAMP levels increase after signaling from Gpr1p and/or Ras1p/2p, cAMP promotes the autophosphorylation of the Tpk subunits (by a yet to be elucidated mechanism) which leads to their dissociation from the regulatory Bcy1p subunits, and the activation of PKA [157,158,159].

Significant differences in signaling phenotype through the cAMP/PKA pathway have been observed between different standard *S. cerevisiae* laboratory strains due to a mutation in *CYR1* [160], which encodes for adenylate cyclase, the protein upon which the signal for both branches of the cAMP/PKA converge on (Figure 2). Strains such as S288c, W303 and Ethanol Red have sequence variants that result in the standard cAMP/PKA signaling response described above. CEN.PK strains on the other hand have the Cyr1p^K1876M^ variant that results in basal constitutive cAMP levels in the presence of d-glucose, rather than the transient d-glucose-induced cAMP pulses of the wild-type protein [160,161]. A consequence of the Cyr1p mutation is that the CEN.PK strains have higher heat tolerance [160], which might contribute to the popularity of this strain background in industrial applications. This variation in signaling response highlights the importance of understanding the signaling not only of *S. cerevisiae,* but also of the specific strain being studied, and complicates comparisons between studies performed in different strain backgrounds.

### 3.4. The Effect of d-Glucose on Other Signaling Pathways

#### 3.4.1. MAPK Pathways: The HOG Pathway and the Filamentous Growth Pathway

Four MAPK pathways in yeast respond to various types of environmental stress and signals, including pheromones, nutrient limitations, osmotic stress and cell wall integrity [51]. Of these four, the highly interconnected HOG and filamentous growth pathways are relevant to sugar signaling. The HOG pathway responds to osmotic stress, for instance caused by increased extracellular sugar concentrations [162] with the Hog1p protein kinase contributing to the induction of the production and accumulation of intracellular glycerol to counteract osmotic stress [163]. The filamentous growth pathway is triggered during nutrient starvation to increase nutrient scavenging and responds to d-glucose starvation via signals from Ras2p [164] and from SNF/Mig1p pathway elements [86].

**Figure 3:**
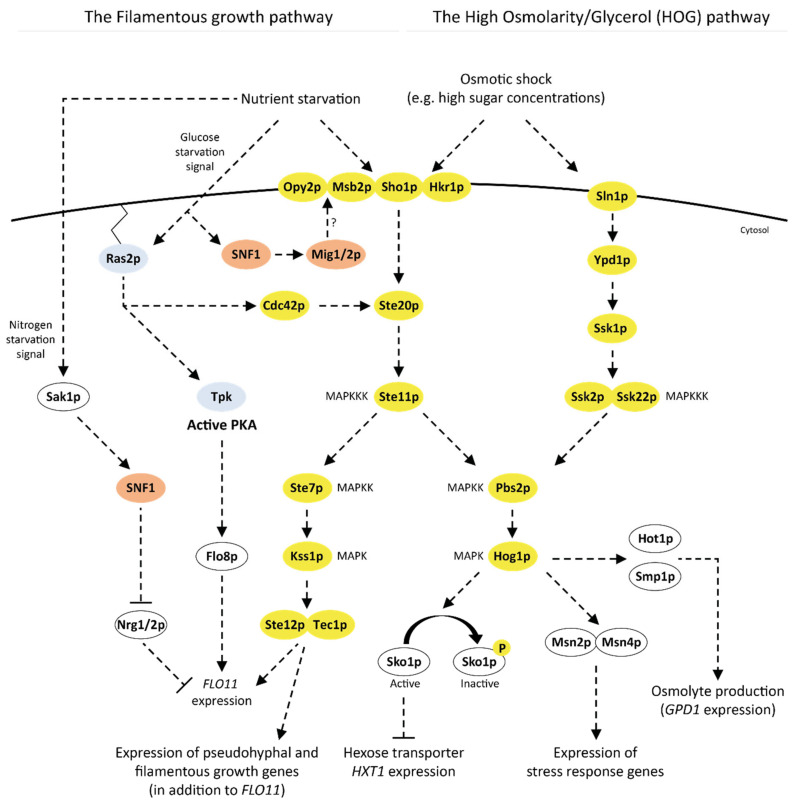
The High osmolarity/glycerol (HOG) pathway and the filamentous growth pathways respond to high d-glucose concentrations (high osmotic stress) and low d-glucose concentrations (nutrient starvation), respectively. The two pathways share many elements, due to Sho1p being involved in sensing both nutrient starvation and osmotic stress. Both pathways belong to the MAPK signaling pathways that transmit the regulatory signals through phosphorylation of target proteins. Note that all the MAPK pathway targets undergo activation and deactivation by phosphorylation, but that only Sko1p has been illustrated with these details in order to facilitate comparison with its activity in Figure 1. MAPK: mitogen activated protein kinase; MAPKK: mitogen activated protein kinase-kinase; MAPKKK: mitogen activated protein kinase-kinase-kinase. Arrows with arrowheads: induction; arrows with hammerheads: repression; dashed arrows: signaling. Yellow shapes: MAPK pathways (HOG1 and filamentous growth pathway); blue shapes: cAMP/PKA pathway elements; orange shapes: SNF1/Mig1p pathway elements; white shapes: proteins from other pathways. Adapted from [85,171,172].

The HOG pathway is controlled by two membrane-bound osmosensors: Snl1p and Sho1p. Although the exact mechanisms of osmosensing are not completely understood [85], the Snl1p branch seems to be controlled by turgor pressure between the cell membrane and the cell wall, as increased extracellular osmolarity results in decreased turgor pressure and pathway activation [85,165,166]. For Sho1p it has been proposed that the sensing of the osmotic stress is achieved through Hkr1p and Msb2p (Figure 3) [167]. In the HOG pathway, the Snl1p and Sho1p-induced signal cascades converge on the activation of Hog1p, which is the final kinase in the pathway and regulator of a number of targets [51] (Figure 3). Apart from the induction of glycerol production genes, Hog1p also regulates the general stress response genes Msn2p/4p [85,163] and is related to sugar signaling through the regulatory effect of Hog1p on the expression of the hexose transporter encoded by *HXT1* during conditions of high osmotic stress, such as high sugar concentrations (Figure 3). While the main regulation of *HXT1* stems from the Snf3p/Rgt2p and SNF1/Mig1p pathways, it is believed that the additional signal from Hog1p serves to increase the d-glucose flux into the cell by upregulating the transporter expression, so that glycerol can be accumulated and osmotic stress can be decreased [168]. It has also been shown that *HXT1* mRNA persisted longer under high osmolarity (40% d-glucose) [169] and that *HXT1* expression was downregulated in a *hog1Δ* strain [170].

During nutrient-limited conditions, yeast cells undergo a change in cell morphology to an elongated filamentous or pseudohyphal state that allows for improved nutrient scavenging and invasive growth on semi-solid substrates [173]. The filamentous growth pathway senses nutrient starvation through a couple of differentiated signals, and to date, three different signal transduction chains have been identified in this pathway (Figure 3): (i) carbon and nitrogen starvation via Sho1p/Msb2p [174,175]; (ii) d-glucose starvation via Ras2p [164,176] and SNF1/Mig1p/2p [86]; and (iii) nitrogen starvation via Sak1p/SNF1 [177,178]. Sho1p has a regulatory role in both the HOG and the filamentous growth pathways and senses nutrient starvation and osmotic stress [86,175,179]. While both Msb2p and Hkr1p are involved in the osmotic stress sensing [167], only Msb2p have been found to be involved in Sho1p sensing of nutrient limitations [174]. Sensing of d-glucose starvation via Ras2p (of the cAMP/PKA pathway) activates filamentous growth in two ways: through signaling to the filamentous growth pathway itself via Cdc42p [164] or via a cascade where the Tpk2p subunit of PKA activates the Flo8p transcription factor which induces filamentous growth genes [176]. Mig1p/2p (two of the targets of SNF1), have also been shown to have a regulatory effect on the filamentous growth pathway during d-glucose limitations, and while several putative mechanisms have been proposed, such as signal transduction to Opy2p and Msb2p, the exact functions have not been elucidated [86]. Finally, it has also been shown that signals from SNF1 and the filamentous growth pathway converge on inducing expression of the filamentous growth gene *FLO11* during d-glucose limitations in haploid *S. cerevisiae* [177]. The authors further suggested that SNF1 is activated in diploid strains during nitrogen limitations rather than during d-glucose limitations [177], which has been supported by a later study showing that SNF1 is activated by the Sak1p protein kinase upon sensing nitrogen starvation [178].

#### 3.4.2. The TOR Pathway

**Figure 4:**
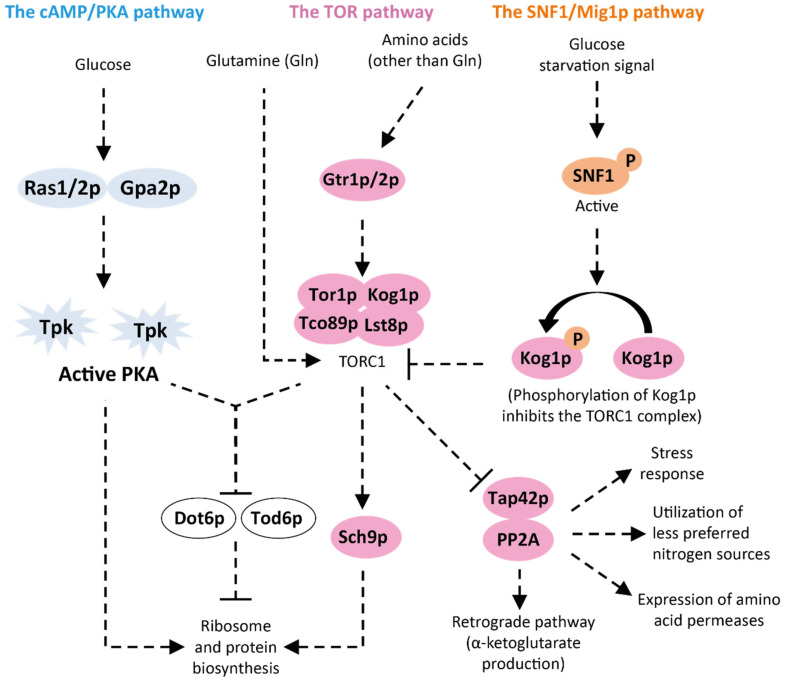
Overview of the *S. cerevisiae* TOR pathway that responds to nutrient availability and controls several biosynthetic and metabolic processes. The TOR pathway senses availability and the type of nitrogen (e.g., amino acids and inorganic nitrogen) and sends signals to induce the transcriptional machinery and nitrogen catabolic pathways. TOR acts in concert with the cAMP/PKA pathway to sense d-glucose and nitrogen availability and, if both signals are present, represses the Dot6p/Tod6p transcriptional machinery repressors. When d-glucose limitation is sensed by the SNF1/Mig1p pathway, cross-talk signals are sent to repress the TOR pathway. Arrows with arrowheads: induction; arrows with hammerheads: repression; dashed arrows: signaling. Blue shapes: cAMP/PKA pathway; pink shapes: TOR pathways; orange shapes: SNF1/Mig1p; white shapes: proteins from other pathways. Adapted from [87,184,189,190].

The *S. cerevisiae* TOR pathway senses nitrogen-availability and is involved in several important cellular activities including ribosome biosynthesis and growth promotion [48,54,180]. The key signaling element of the pathway is the TOR complex 1 (TORC1) kinase, which consists of Tor1p/2p serine/threonine kinases complexed with Kog1p, Tco89p, and Lst8p (Figure 4). TORC1 is activated upon sensing of intracellular amino acids (Figure 4): either by direct interactions of TORC1 and glutamine, the preferred nitrogen source of *S. cerevisiae* [181], or by Gtr1p/2p and their co-sensors after sensing of other amino acids [54,182]. TORC1 transduces signals to two downstream regulatory elements: Sch9p and the Tap42p-PP2A (protein phosphatase 2A) complex that in turn regulate several different targets [48,182]. Sch9p is activated by TORC1 when nitrogen is available and induces the expression of genes for protein and ribosome biosynthesis. Tap42p-PP2A is activated upon nitrogen starvation and activates TFs that induce the expression of genes for amino acid transporters, stress response, utilization of nitrogen sources other than ammonium, and the retrograde pathway that catalyzes the formation of α-ketoglutarate from the TCA which subsequently can be used to synthesize glutamine [48,54,182,183,184] (Figure 4). While the TOR pathway is involved in nitrogen sensing [48], additional parallel mechanisms have evolved to respond to different nitrogen-availability cases [185], which indicates a level of complexity of nitrogen sensing in *S. cerevisiae* that is beyond the scope of the current review.

In addition to its role in nitrogen sensing, the TOR pathway has also been associated with sugar metabolism due to its cross-talk with the sugar signaling routes [186]. For example, d-glucose is sensed by cAMP/PKA and nitrogen availability by TOR [87,134], but both pathway signals converge to inactivate the Dot6p/Tod6p repressors controlling the ribosome regulon (Figure 4) [187]. The two signaling pathways can act in parallel or together to control the expression of the same set of target genes; however, cAMP/PKA controls gene expression during transitions in and out of growth, whereas TOR controls steady-state expression [87]. Meta-analysis of two previous transcriptomics studies have indicated that TOR co-regulates up to 58% of the d-glucose-responsive genes in *S. cerevisiae* [188].

Whether d-glucose can be sensed by the *S. cerevisiae* TOR pathway or not is still under debate. A current hypothesis suggests that SNF1 deactivates TORC1 by phosphorylation of the Kog1p subunit during d-glucose starvation [184,190], i.e., the d-glucose sensing is achieved via SNF1/Mig1p pathway cross-talk (Figure 4). A similar activity has been found in mammals, where the Snf1p ortholog AMPK inhibits mammalian TORC1 in the absence of d-glucose [54]. Cross-talk between the TOR pathway and the sugar sensing pathways has also been observed via Sch9p that controls expression of many target genes of the TOR pathway [191,192]. SNF1 has been shown to phosphorylate Sch9p during signaling from the intrinsic aging defense pathway in a TORC1-independent manner [193], and it is possible that the TORC1-dependent and TORC1-independent d-glucose-responses of Sch9p are communicated by SNF1 in response to d-glucose signals. Furthermore, Hog1p has been found to have an inhibitory effect on the TOR pathway during osmotic stress [184].

#### 3.4.3. The Galactose Regulon

As mentioned, *S. cerevisiae* prefers d-glucose over any other carbon source, and the expression of genes required for metabolism of alternative carbon sources is avoided through CCR. In the absence of d-glucose, however, *S. cerevisiae* maintains the capacity to utilize other natural carbon sources, such as the hexose sugar d-galactose.

The *GAL* regulon of *S. cerevisiae* controls expression of the enzymes required for assimilation of d-galactose and its regulatory mechanisms have been highly characterized [89,194]. After transport into the cell, via the Gal2p permease, d-galactose is shuttled into glycolysis at the level of glucose-6-phosphate through the actions of galactokinase (Gal1p), transferase (Gal7p), epimerase (Gal10p) and mutase (Pgm2p). The genes encoding these enzymes (*PGM2* excepted) belong to the *GAL* regulon that also includes *GAL3*, *GAL4* and *GAL80*, all encoding regulatory proteins. The expression of these *GAL* genes is governed by two main TFs: the Mig1p repressor and the Gal4p activator. All *GAL* gene promoters contain recognition sequences for both TFs but the overall transcriptional state depends on the carbon sources being sensed. In the presence of d-glucose, Mig1p efficiently blocks transcription regardless of whether d-galactose is present or not. In the absence of both d-glucose and d-galactose, repression by Mig1p is relieved but the *GAL* genes are still not expressed due to the coregulator Gal80p, which interacts with Gal4p, preventing recruitment of the transcriptional machinery. The *GAL* genes are ultimately induced upon addition of d-galactose through the action of the third regulatory protein, Gal3p. Gal3p is a paralog of Gal1p, but appears to have lost its galactokinase activity [195]. Instead, it senses d-galactose by a yet not fully elucidated mechanism and interacts with Gal80p to prevent its inhibition of Gal4p, and thereby allowing for transcription of the *GAL* genes. The mechanism behind the interaction between Gal3p and Gal80p is not yet fully understood, but subcellular sequestering has been proposed as (i) Gal3p is solely found in the cytoplasm; and (ii) Gal80p changes its subcellular localization from the nucleus to the cytoplasm upon addition of d-galactose.

Deletion of *GAL3* prevents the rapid (within minutes) induction of *GAL* genes seen upon addition of d-galactose to non-repressing media. However, strains containing *gal3*Δ display induced *GAL* genes within 2–5 days, a phenomenon called long-term adaptation [196]. During in vitro transcription experiments Gal1p was shown to substitute for Gal3p to produce rapid d-galactose induction of *GAL* genes, but only at a protein concentration 40 times higher than that of Gal3p [197].

Apart from the *GAL* genes, a few genes were found to be regulated by Gal4p, Gal80p and Gal3p: *MTH1*, whose product represses genes encoding hexose transporters other than Gal2p, *PCL10*, encoding an activator and target specifier of the Pho85p cyclin-dependent kinase, leading to reduced glycogen synthase Gsy2p activity in the presence of d-galactose, and *FUR4*, encoding an uracil permease [93,198,199,200]. In addition, the 17 base pair recognition site for Gal4p has been found in around 200 loci in the *S. cerevisiae* genome but it is possible that the majority of these are not available for Gal4p due to the DNA being inaccessible within nucleosomes [201].

Due to the tight pathway regulation, *GAL* promoters have been widely used for inducible expression of heterologous genes. More specifically, the d-glucose repressible *GAL1*p and *GAL10*p are cornerstones of the *S. cerevisiae* genetic toolbox [202,203] and are used for inducible expression of genes.

### 3.5. Cross-Talk between the Different Sugar Signaling Pathways

As outlined above, signaling pathways have self-regulatory mechanisms in order to respond to variations in the available levels of d-glucose and other sugars by turning parts of the network on or off upon different signals (Figure 2). As an additional layer of control, signaling elements from different pathways can interact with each other at the cascade level and on the gene expression/TF level (Figure 2). This cross-talk and pathway co-regulation adds an increased degree of redundancy to the network that enhances the specificity in the signaling response and protects the pathway functionality from e.g., the effect of loss-of-function mutations.

One of the most prominent examples of signaling cross-talk is how the expression of hexose transporters is influenced by the cAMP/PKA, SNF1/Mig1p and HOG pathways, in addition to the direct control elicited by the Snf3p/Rgt2p pathway. Sensing of d-glucose by the Snf3p/Rgt2p transmembrane sensors results in degradation of the Rgt1p co-repressors Mth1p and Std1p [204] which leaves Rgt1p open to phosphorylation by PKA (Figure 2), a response that has also been found to be activated by the sensing of d-glucose signal by the cAMP/PKA pathway [106]. Signals for d-glucose repression of Mth1p are also controlled through the SNF1/Mig1p pathway [114]. Upon phosphorylation by PKA, Rgt1p is converted from a transcriptional repressor to an activator and the degree of phosphorylation regulates the strength of its effect as an activator. Low d-glucose concentrations (~1 g L^−1^) lead to low PKA activity while only a few Rgt1p sites are phosphorylated, which is sufficient for *HXT2/4* induction [170]. High d-glucose concentrations lead to hyperphosphorylation of Rgt1p that is strong enough to induce *HXT1* [105], and simultaneously, the repression of *HXT2/4* by Mig1p/2p [107]. Mig1p/2p dephosphorylation is required for translocation into the nucleus and subsequent repression of gene expression, and for Mig1p this is controlled by the sensing of d-glucose by the SNF1/Mig1p pathway [205].

Other examples of cross-talk within the sugar signaling pathways include the following: (i) PKA negatively regulates SNF1, but SNF1 can phosphorylate Cyr1p which leads to diminished PKA activity [148]; (ii) the *GAL* regulon can only be induced when two separate signals are sensed: presence of d-galactose and absence of d-glucose (relief of d-glucose catabolite repression [88]); and (iii) SNF1 and PKA each induce the general stress response TF Msn2p and control its nuclear translocation by phosphorylation/dephosphorylation [206,207]. SNF1 cross-talk in *S. cerevisiae* has been the main subject of reviews in the past [205,208]. While the fundamental mechanisms of these pathways have been established decades ago, new interactions are still being discovered [148,205], and the degree of cross-talk might be even higher than we currently know.

### 3.6. Connections between Sugar Signaling and Glycolysis

The major sugar signaling pathways throughout Section 3 were governed by a d-glucose signal. This signal can be categorized as extracellular and intracellular d-glucose signals, with the former affecting the Snf3p, Rgt2p and Gpr1p sensors and the latter the SNF1/Mig1p pathway and the Ras1p/2p branch of the cAMP/PKA pathway. Whereas the extracellular signal is triggered by d-glucose, and to some extent its closely related analogues, the intracellular signal can originate from various intracellular changes. These include changes in protein phosphorylation and ubiquitination, as described above, but also changes in the level of intracellular metabolites formed during sugar metabolism. The signaling effects of intracellular metabolites is not as well understood as the extracellular d-glucose signals, but the knowledge in this field is expanding. Below is a summary of key reported examples of signaling-glycolysis interactions.

Intracellular signaling through the SNF1/Mig1p pathway and the Ras1p/2p branch of the cAMP/PKA pathway has long been known to be dependent on d-glucose uptake and phosphorylation, that is, formation of the glycolytic intermediate glucose-6-phosphate [116,155]. The signal does not require any specific sugar transporter or glucose kinase; however, d-glucose repression of certain genes (e.g., *SUC2* and *GAL*) is dependent on the regulatory function of Hxk2p [116]. Glucose-6-phosphate has also been proposed to be involved in the regulation of d-glucose repression through SNF1/Mig1p. This has been suggested since neither limiting the glycolytic step after glucose-6-phosphate isomerization nor adding the d-glucose analogue 2-deoxy-d-glucose (which can also be phosphorylated, but not further metabolized) changed the native d-glucose repression response [116,123]. Likewise, there are indications that trehalose-6-phosphate, the precursor of the storage carbohydrate trehalose, which is known to have a signaling function in plants [209,210], has an inhibitory activity on SNF1 in *S. cerevisiae,* but the exact mechanisms remain to be elucidated [211]. The intracellular d-glucose signal affecting the Ras1p/2p branch of the cAMP/PKA pathway, on the other hand, appears to also originate from the glycolysis (Figure 2). Peeters and colleagues were able to demonstrate that the glycolytic intermediates fructose-1,6-bisphosphate and, to lesser extents, dihydroxyacetone-3-phosphate and glyceraldehyde-3-phosphate can affect the activity of Ras1p/2p, probably by activating Cdc25p (Figure 2) [212].

Fluxes of glycolytic intermediates have also been used as indicators of overall metabolic state of the cell. For example, van Heerden and colleagues studied cases where a yeast mutant with a deletion in *TPS1*, encoding a trehalose-6-phosphate synthase subunit, would fail to initiate a steady-state flux through glycolysis upon addition of d-glucose to a d-galactose culture, instead entering an imbalanced state [213]. The authors found that (i) the imbalanced state also occurs in a small subpopulation of wild-type yeast; and (ii) both states could be reached in silico using kinetic modeling with slight random modifications to initial enzyme and metabolite concentrations. They concluded that the dynamic nature of the potential metabolic states reachable during the glycolytic start-up would require a robust regulatory network that is responsive to metabolite fluxes for the yeast to reliantly end up in the balanced glycolytic state each new time the cell starts the glycolysis up anew. However, the authors did not investigate the mechanisms behind the proposed regulation [213].

## 4. What Happens on d-Xylose, and Why?

### 4.1. d-Xylose Signaling in Natural and Engineered S. cerevisiae

As reviewed in Section 3, the sensing and regulation of d-glucose catabolism is ensured by several complex and interconnected mechanisms involving molecular control at the gene and protein levels. However, the response of these pathways to a non-natural carbon source such as d-xylose is expected to differ. Whether engineered *S. cerevisiae* can sense the d-xylose sugar itself and, in extension, if it can sense it as a metabolizable sugar has long been debated [35,37,38,214,215,216] and the current results are ambiguous. The starvation response, expression of genes and activation of enzymes related to respiratory growth and gluconeogenesis (exemplified in Table 3 for XR/XDH strains), and partial activation of CCR suggest that *S. cerevisiae* does not sense d-xylose as a fermentable sugar [34,35,37,214,216,217]. On the other hand, partial CCR de-repression on d-xylose and similarities in adenylate energy charges (a measurement of the energetic availability of the cell, defined as (ATP + ½ADP)/(ATP + ADP + AMP) [218]) between d-xylose and d-glucose implies that it does affect the signaling [215,219,220]. In the present section, we discuss the known and putative effects of d-xylose on sugar signaling routes in *S. cerevisiae* strains that have or have not been engineered for d-xylose utilization.

#### 4.1.1. The Snf3p/Rgt2p Pathway Weakly Senses d-Xylose

**Figure 5:**
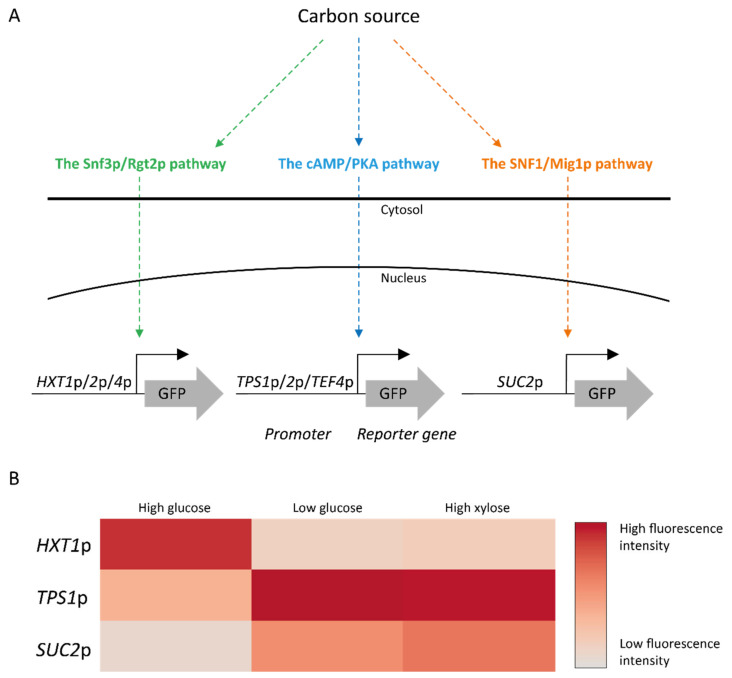
Schematic view of the biosensors constructed by Brink et al. [194] and the comparison of the effect of high d-glucose, low d-glucose and high d-xylose condition in the biosensors (heat map). (**A**). Fluorescent biosensors were constructed to assay the transcriptional effect of the three main sugar signaling pathways in the presence of d-xylose or d-glucose by coupling the promoters of signaling pathway target genes with a green fluorescent protein (GFP). (**B**). By following and quantifying the fluorescence intensity of the biosensor strains over time with flow cytometry, the known repression and induction conditions of the chosen promoters during presence of d-glucose were confirmed [107,224,225,226] and subsequently used to analyze the response to d-xylose in XR/XDH engineering strains [77,222].

Despite the only difference between d-xylose and d-glucose being one additional hydroxymethyl-group for the latter, d-xylose has been reported not to trigger the Snf3p d-glucose sensor in *S. cerevisiae* nor the Mth1p degradation in a *rgt2*Δ strain [221]. However, Dietvorst and colleagues used only qualitative SDS-PAGE which might lack the resolution required to detect weak signaling effects. Indeed, a later study revisited the conclusions using fluorescent biosensors where the promoters of signaling pathway target genes, such as *HXT1/2/4* for the Snf3p/Rgt2p pathway, were coupled to a green fluorescent protein (GFP) (Figure 5) [222]. In biosensor strains an engineered XR/XDH pathway, *HXT1*p-GFP was induced by high levels of d-glucose but repressed by d-xylose, *HXT2*p-GFP and *HXT4*p-GFP were induced by low concentrations of d-glucose but also by high concentrations of d-xylose (Figure 5) [77]. A similar pattern of d-xylose gene induction was also observed in a transcriptomic study on XR/XDH strains: *HXT1* was not induced whereas *HXT2* showed signs of being both up- and downregulated by the pentose sugar [217]. Several studies have since reported the partial induction of *HXT2* by d-xylose [77,222,223].

In the biosensor strains, the GFP signal on d-xylose for these two genes was distributed over two populations (one repressed and one induced). This was seen both for strains that had not been engineered with a d-xylose-utilization pathway [222], and after a transporter with increased d-xylose specificity was added to the same strains [77]. Similar results were also reported by Wu and co-workers, who detected partial *HXT2* induction with real-time quantitative PCR in non-xylose utilizing strains and linked this d-xylose signal to Snf3p, the sensor for low d-glucose concentrations [223]. Taken together, the partial *HXT2* induction in both engineered and non-engineered strains indicates that the Snf3p/Rgt2p pathway does indeed sense extracellular d-xylose. However, it remains unclear whether intracellular d-xylose or d-xylose-derived metabolites also affect this signaling pathway.

#### 4.1.2. d-Xylose Affects the SNF1/Mig1p Pathway Both Directly and Indirectly

d-Xylose has been shown to affect the SNF1/Mig1p pathway in different ways, e.g., by direct interactions with proteins of the pathway and by altering the expression of genes under control of the pathway. d-Xylose notably triggers an irreversible Hxk2p autophosphorylation at Ser158 [227,228,229], which is a different site than the Ser15 that the SNF1 complex phosphorylates in the regular SNF1/Mig1p pathway [118]. The inactivation caused by phosphorylation of Ser158 impairs the catalytic activity of Hxk2p and decreases the rate of the first step in glycolysis, i.e., the phosphorylation of d-glucose to glucose-6-phosphate (Figure 2). The SNF1 subunit Snf1p, responsible for the catalytic activity of the complex, is allosterically regulated by the ADP:AMP ratios, and SNF1 is more resistant to inactivation by Glc7p during low ADP:AMP ratios [128]. However, the cellular adenylate energy charge has been found to be similar during high concentrations of d-xylose or d-glucose [215,220], which suggests that the activity of SNF1 may not be affected by d-xylose.

One of the genes under control of the SNF1/Mig1p pathway, *SUC2,* has a long history as a sensor for d-glucose repression [230,231,232,233]. The gene encodes invertase, a usually secreted protein that splits the disaccharide sucrose into d-glucose and d-fructose monosaccharides by hydrolysis [234]. *SUC2* has an unusual expression pattern as it is repressed both on high d-glucose concentrations and in the absence of d-glucose and is only induced during low d-glucose conditions (0.5–5 g L^−1^) [224]. When using a biosensor with *SUC2* driving GFP expression, 25–100 g L^−1^ d-xylose did affect the fluorescent signal in a non-xylose engineered *S. cerevisiae* strain; however, a mixture of 5 g L^−1^ of d-glucose and 50 g L^−1^ d-xylose led to a 150% increase in GFP signal compared to that of 5 g L^−1^ of d-glucose without any d-xylose [222]. Furthermore, when the same biosensor was implemented in a XR/XDH strain, GFP was induced by both high and low levels of d-xylose and the cumulative effect during 5 g L^−1^ of d-glucose and 50 g L^−1^ d-xylose administration was no longer observed [77]. Since *SUC2* is induced only during low levels of d-glucose [224], the biosensor results suggested that high concentrations of d-xylose are sensed by *S. cerevisiae* as if it was sensing low concentrations of d-glucose [77]. The d-xylose induction in the non-engineered [222] and in the engineered strains [77,235] indicate that both the d-xylose molecule itself and some of its intracellular metabolites are sensed by the SNF1/Mig1p pathway.

An early example of d-xylose signaling engineering in the SNF1/Mig1p pathway by Roca and colleagues (2004) could demonstrate an increased d-xylose consumption rate in *mig1*Δ and *mig1*Δ *mig2*Δ strains [236]. The authors attributed this to a downregulation of the CCR, but suggested that CCR was a secondary issue in d-xylose utilization that should be addressed once consumption rates have been increased [236]. Today, with the added knowledge of almost two additional decades of research into d-xylose engineering, signaling targets such as these emerge as more important than ever.

#### 4.1.3. Assimilation of d-Xylose Is Weakly Sensed by the Intracellular Branch of the cAMP/PKA Pathway

The results from several studies point towards a lower degree of cAMP/PKA signaling of d-xylose fermenting cells, resulting from no extracellular sensing and/or poor intracellular activation [77,223,237]. For instance, contrary to its response to d-glucose, the extracellular sensor Gpr1p (Figure 2) did not trigger a cAMP spike in the presence of d-xylose in a non-xylose-engineered strain [237]. Similar results were found when using GFP biosensors coupled to promoters from the PKA-activated trehalose pathway (*TPS1*p-GFP and *TPS2*p-GFP), with no change in expression of these sensors in the presence of d-xylose in non-engineered strains [222]. However, in XR/XDH-engineered strains grown on d-xylose, signaling was reported by several independent studies: genes related to trehalose were found to be expressed on d-xylose [37] and the activity of trehalase on d-xylose was found to be only 35% of the activity measured on d-glucose [223]. Furthermore, high levels of d-xylose (50 g L^−1^) induced GFP-based biosensors reporting on the cAMP/PKA signaling, albeit only to a similar level as under low d-glucose conditions (1 g L^−1^ d-glucose) [77].

**Figure 6:**
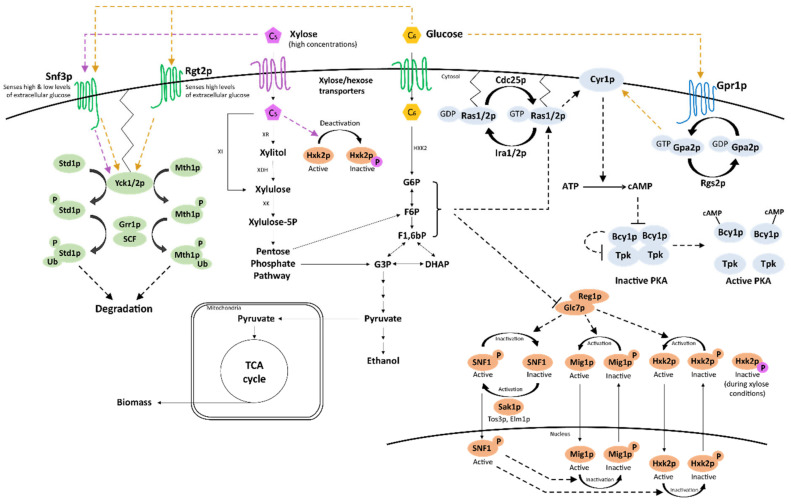
Model of d-xylose sensing and signaling in *S. cerevisiae* compared to known d-glucose signaling events. d-Xylose may be sensed by Snf3p, but not by Rgt2p or Gpr1p. d-Xylose metabolism has been shown to result in a decreased glycolytic flux when compared to d-glucose [238,239], which in turn will affect the concentration and signaling strength of the glycolytic intermediates G6P (glucose-6-phosphate), F6P (fructose-6-phosphate) and F1,6bP (fructose-1,6-bis-phosphate). Hxk2p is phosphorylated in two different positions depending on the origin of the signal: phosphorylated in Ser15 by SNF1, and autophosphorylated in Ser158, triggered by absence of d-glucose and presence of d-xylose, respectively [36]. Dashed purple arrows: d-xylose signaling; dashed orange arrows: d-glucose signaling; black dashed arrows: downstream signaling events (irrespective of sugar stimulus); arrows with arrowheads: induction or activation; arrows with hammerheads: repression or deactivation; solid arrows: metabolic or transport reactions; circles with P: phosphorylations (the color of the circle indicate which signaling pathway controls the phosphorylation); purple pentagons: d-xylose; yellow hexagons: d-glucose. Green shapes: Snf3p/Rgt2p pathway; blue shapes: cAMP/PKA pathway; orange shapes: cAMP/PKA pathway. Partially adapted from [79].

As discussed in Section 3.6, it has been proposed that the Ras1p/2p branch of the cAMP/PKA pathway senses intracellular d-glucose-derived signals through concentrations, rates and/or ratios of a few glycolytic metabolites [156,212]. Therefore, it may be hypothesized that any differences in cAMP/PKA signaling between d-xylose and d-glucose could be related to the slower catabolism of d-xylose that leads to lower concentrations of glycolytic intermediates, and possibly to levels comparable to that of the presence of low concentrations of d-glucose. Indeed, cultivations of engineered strains on d-xylose have been found to result in a decreased flux through glycolysis compared to d-glucose cultivations [238,239]. This indicates that the signal strength to the intracellular cAMP/PKA branch might be weaker when cells are grown on d-xylose rather than on d-glucose (Figure 6), which is consistent with the above suggestions that cAMP/PKA activity is lower in d-xylose cultures. Further indications of a decreased signal intensity through the cAMP/PKA pathway on d-xylose has been suggested by measuring the balance between the concentrations of GTP, GDP and GMP, as both branches of the cAMP/PKA pathway rely on G-proteins (Ras1p/2p and Gpa2p respectively) that are activated upon binding of GTP [155,240]. Different types of carbon sources have been shown to affect the GTP:GDP:GMP ratio in the cell: GTP and GDP accumulated during growth on 20 g L^−1^ d-glucose in a non-engineered *S. cerevisiae* strain (to a GTP:GDP:GMP ratio of 3:0.9:0.1); when the same non-engineered strain was cultivated on 20 g L^−1^ d-xylose, GTP was undetectable and total GMP increased (GTP:GDP:GMP ratio of 0:0.2:1.5) [241]. The authors also assayed an XR/XDH strain growing on 20 g L^−1^ d-xylose and found that the recombinant pathway clearly tilted the ratio in favor of GTP (GTP:GDP:GMP ratio of 1.7:0.9:0.8) compared to the non-engineered strain on d-xylose, but not as much as when cultured in d-glucose [241].

#### 4.1.4. Effect of d-Xylose on Other d-Glucose-Responsive Signaling Pathways

As was discussed in Section 3.4, d-glucose affects the signaling in more pathways than just the three main sugar signaling networks. The effects of d-xylose on the HOG, filamentous growth, TOR and *GAL* pathways are however less investigated than the effects of d-glucose. Below we summarize the current understanding of the connection of these four pathways to d-xylose utilization and signaling.

Studies on the *S. cerevisiae* HOG pathway often use high concentrations of d-glucose or NaCl to induce osmotic stress [242], and while studies using other sugars are scarce [243], a high concentration of sugar is likely to contribute to osmotic stress regardless of sugar type. However, the affinity for d-xylose among native hexose transporters is up to 200 times lower than for d-glucose [22]. Therefore, d-xylose molar concentrations three times higher than that of d-glucose (corresponding to 50 g L^−1^ d-xylose vs 20 g L^−1^ d-glucose) have commonly been used for d-xylose fermentations with engineered strains to drive d-xylose uptake [27,236,244,245]. This implies that the osmotic stress must be higher in the 50 g L^−1^ d-xylose cultivations compared to the standard 20 g L^−1^ d-glucose cultures, and that a different degree of HOG signaling might be expected between these two typical sugar loads. *GRE3* encodes an aldose reductase that is a common deletion target in d-xylose engineered strains in order to reduce xylitol formation [246]. *GRE3* is induced by the HOG pathway upon osmotic stress [247], and the necessity of its deletion to reduce xylitol by-product formation is highlighted by how HOG is likely to become activated at high d-xylose concentrations.

A handful of studies have investigated the effect of d-xylose on the HOG pathway. A heterozygous premature stop codon in the MAPKKK gene *SSK2* of the HOG pathway was found to be linked to increased d-xylose consumption rates in an engineered *S. cerevisiae* strain that had undergone adaptive laboratory evolution, and the phenotype was confirmed after introducing *ssk2*Δ into the parental strain [248]. A similar laboratory evolution experiment led to the isolation of a strain with improved capacity for anaerobic d-xylose fermentation that, among other sequence variants, was found to have a *HOG1* loss-of-function mutation [249]. In a follow-up study, the degree of phosphorylation of enzymes in the glycolysis and trehalose pathways were found to be lower in a *hog1*Δ strain during d-xylose consumption, compared to a strain without the deletion [250]. As has been pointed out by Wagner and colleagues, the HOG and cAMP/PKA pathways have opposing functions, with HOG inducing stress response genes via Msn2p/4p and cAMP/PKA repressing the same genes while also promoting growth [250]. The elevated osmotic stress, likely induced by 50 g L^−1^ d-xylose compared to 20 g L^−1^ d-glucose, could therefore result in a stress signal that diminishes the effect of the induction of growth-promoting genes by PKA. The link between HOG and *HXT1* expression also indicate that the HOG pathway plays a role in sugar signaling [170], but whether d-xylose results in a different signal to *HXT1* than d-glucose has to our knowledge not been investigated.

The filamentous growth pathway is induced during growth on non-fermentable carbon sources [173], and while studies have suggested that engineered *S. cerevisiae* senses d-xylose as a non-fermentable carbon source [35,37,38,214,215], few studies have investigated the effect of d-xylose on the filamentous growth pathway. One of the studies on this topic found that d-xylose did not inhibit expression of a *FLO11* gene variant found in an industrial self-flocculating strain (a protoplast fusion of *S. cerevisiae* and *Schizosaccharomyces pombe*), whereas shifting to sucrose, maltose, and mannose led to an increased inhibitory effect [251]. Whether these findings also apply for the regular *S. cerevisiae FLO11* gene remains unknown. Very little is also known about whether d-xylose results in a different signal in the TOR pathway compared to d-glucose, but mutations in genes that regulate the TOR pathway have been found in improved XI strains, including *PMR1* [20,252] and *SAP190* [249]. Since cross-talk with the cAMP/PKA and SNF1/Mig1p pathways has been established for both the filamentous growth and TOR pathways to trigger nutrient scavenging during nutrient limitations and to regulate growth promotion during nutrient availability, respectively, there is a possibility that d-xylose affects the signaling in these pathways differently to d-glucose. This remains to be tested in future studies.

Various genes from the galactose pathway have been used to improve d-xylose utilization. For instance, the galactose transporter Gal2p, the expression of which is controlled at the gene level by the *GAL* regulon, has been shown to transport d-xylose [111,253] and xylitol [254] Gal2p variants have been used in several studies to increase utilization of d-xylose by improving its transport inside the cell [255,256]. Phosphoglucomutase is encoded by *PGM2* and catalyzes the interconversion between glucose-1-phosphate and glucose-6-phosphate, which is the last step in the Leloir pathway. Overexpression of *PGM2* has been shown to improve both d-galactose [257] and d-xylose utilization in an XR/XDH strain [258], which shows that there are links between d-galactose and d-xylose metabolism [37,72]. d-Xylose has also been observed to affect the d-galactose metabolism at a regulatory level, as transcriptome analysis of XR/XDH strains found that *GAL1/3/4/7/10* were upregulated on d-xylose compared to [37,72]. The Gal3p protein, which is one of the signal transducers in the GAL regulon, has been shown to respond to d-xylose (albeit with a less strong response compared to its primary sugar, d-galactose) [259]. Gal3p variants with higher d-xylose sensitivity have been generated (discussed further in Section 5.2 below).

The decreased glycolytic flux during d-xylose cultivations has also been suggested to result in redox imbalances [220]. However, while energy-related cofactors such as GTP and ATP have documented effects on the cAMP/PKA and SNF1/Mig1p pathways [128,215,220,241], as discussed in Section 3.3 and Section 4.1.2, respectively, the impact of NAD(P)H/NAD(P)^+^ ratio on the signaling pathways is less understood and the mechanisms of how the cell senses redox imbalances in general is yet to be elucidated.

#### 4.1.5. Proposed Mechanisms for d-Xylose Sensing

The studies reviewed above have shown that d-xylose affects signaling pathway elements in wild-type and engineered *S. cerevisiae* in several ways. Based on the studies discussed in Section 4.1 and the known d-glucose signaling mechanisms (Section 3), we propose four different, possibly overlapping, explanation models as to how d-xylose affects *S. cerevisiae:* (i) d-xylose itself is partially sensed as a utilizable carbon source by some of the signaling pathways; (ii) the structural similarity between d-xylose and d-glucose allows d-xylose to be sensed by non-d-xylose specific mechanisms (such as d-glucose sensors) but results in different signal strengths than those resulting from sensing of d-glucose due to their minor differences (a single hydroxymethyl group); (iii) glycolytic intermediates and other metabolic intermediates shared between d-glucose and d-xylose metabolism (such as glycolysis, gluconeogenesis and the pentose phosphate pathway) are sensed by the cell, and the differences in their levels and formation rates from d-xylose compared to d-glucose results in different signal strengths; and (iv) the differences in cofactor demand and formation during d-xylose assimilation (e.g., redox and energy carriers) compared to the preferred carbon source d-glucose is sensed as a change in the cellular homeostasis, which limits the cell from entering its full fermentation or respiration states. While the current understanding of d-xylose signaling in *S. cerevisiae* does not rule out the possibility that there are additional explanation models than these four, they point towards future directions of fundamental and applied studies on *S. cerevisiae* d-xylose utilization.

### 4.2. d-Xylose Signaling in Other Xylose-Utilizing Species

While *S. cerevisiae* cannot naturally use d-xylose, the sugar is used by a variety of yeast and bacterial species. In this section, we discuss mechanisms that have been associated with the regulation of d-xylose metabolism in pentose-utilizing yeast and bacterial species. A particular focus is put on CCR and on the bacterial d-xylose regulators (XylR) that can either induce (referred below as XylR-I) or repress (referred below as XylR-R) genes involved in d-xylose utilization. These examples show that d-xylose signaling might differ from species to species, and that the knowledge acquired from other microbes can be used both to shed light on the *S. cerevisiae* signaling mechanisms and to develop novel engineering strategies (as will be discussed in Section 5.2 below).

#### 4.2.1. d-Xylose Regulation in Other Yeast Species

*Scheffersomyces stipitis* represents a pioneer yeast in the study of d-xylose fermentation to ethanol [260]. In this Crabtree-negative yeast, metabolism is tightly connected to the oxygenation level: with respiration under aerobic conditions, fermentation under low oxygen levels and no metabolic activity under anaerobiosis [261,262]. In *Sc. stipitis*, both d-glucose and d-xylose are used efficiently, and similar ethanol yields and growth rates have been reported under microaerobic conditions [263]. This, and the fact that *Sc. stipitis* XR uses both NADH and NADPH as cofactors, has led to the introduction of its XR/XDH genes for d-xylose utilization in several organisms, including *S. cerevisiae* [18]. Drawbacks of *Sc. stipitis*, however, include the requirement for precise oxygenation conditions [264], d-glucose repression of d-xylose utilization and a limited tolerance to ethanol and to inhibitors present in lignocellulosic hydrolysates [265,266,267].

There is limited information about the mechanisms governing sugar sensing in *Sc. stipitis.* Homology search has identified conserved sequences for key regulatory proteins found in *S. cerevisiae,* such as Snf1p, Snf3p, Grr1p and Mig2p, but it failed to find other important regulatory elements such as Mig1p and Rgt1p [268]. Interestingly, it has been shown that the response observed in *S. cerevisiae* on d-glucose corresponds to how *Sc. stipitis* responds to oxygenation, and that the repression of d-xylose utilization by d-glucose can be relieved by limiting respiration [269]. Furthermore, homologs of Snf3p and Sks1p responding to d-glucose in *S. cerevisiae* instead respond to oxygen level in *Sc. stipitis* [268,270].

Recently, focus has moved towards the study of the *Spathaspora* clade as several species, notably *Sp. passalidarum*, have shown the ability to ferment d-xylose to ethanol under anaerobic conditions [271]. This property has been attributed to the presence of the *XYL1.2* gene encoding an XR with increased affinity for NADH [44]. However, *Sp. passalidarum* also presents a peculiar behaviour regarding CCR: although d-xylose is used after d-glucose, d-xylose utilization is not inhibited by the presence of the d-glucose analog 2-deoxy-d-glucose, hinting at the presence of a non-canonical signaling mechanism [272]. Clearly, increasing knowledge on sugar sensing and signaling in these and other non-conventional yeast species could help advancing the engineering of *S. cerevisiae* d-xylose sensing.

#### 4.2.2. XylR as an Inducer of the d-Xylose Operon (XylR-I)

The bacterium *Escherichia coli* represents one of the most popular industrial cell factories and can efficiently utilize d-xylose and d-glucose under both aerobic and anaerobic conditions, although it displays lower growth rates and biomass yields on d-xylose [273]. In mixed sugar cultivations, CCR prevents d-xylose to be used when d-glucose is present in the medium [274]. In the absence of d-glucose, activation of the d-xylose pathways requires d-xylose to be sensed by XylR, a protein sharing similarities with the LacI-repressor family [275]. d-Xylose binding induces a change in the conformation of XylR that enables the protein to bind to the promoter region of the xylose operons *xylAB* and *xylFGHR*, and induce their expression [275,276]. It has been shown that mutations in XylR-I (variants R121C and P363S) could relieve the CCR and induce co-consumption of d-glucose and d-xylose [277], highlighting the key role of XylR for d-xylose pathway activation.

#### 4.2.3. XylR as a Repressor of the Xylose Operon (XylR-R)

One example of repression by XylR (XylR-R) can be found in *Caulobacter crescentus* that utilizes d-xylose via the oxidative Weimberg pathway. In this bacterium, XylR is also the major regulator governing d-xylose catabolism (via the *xylXABCD* operon) and transport (via the *xylE* operon). However, in contrast to *E. coli*, *C. crescentus* XylR functions as a transcriptional repressor (XylR-R): in the absence of d-xylose, XylR-R binds to the promoters of the *xyl* operons, preventing transcription; when d-xylose is sensed, XylR-R is released and transcription proceeds [278]. Interestingly, similar induction of the promoter controlling xylose genes was found on d-xylose and on mixture of d-xylose and d-glucose [279], indicating that the d-xylose pathway in *C. crescentus* is not under CCR. At least fifty-one genes were found to be induced on d-xylose as compared to d-glucose [280]; in addition to the expected *xylXABCD* and *xylE* operons, eight genes coded for polysaccharide-degrading enzymes and secreted proteins, and ten genes encoded receptors and transporters. This hints towards a co-induction of genes involved in the utilization of d-xylose and sugar polymers found in lignocellulosic biomass. XylR-R systems have also been found in e.g., *Bacillus subtilis*, *Lactobacillus pentosus* and *Staphylococcus xylosus* [281,282,283].

## 5. Current Status of Engineering of *S. cerevisiae* d-Xylose Signaling

### 5.1. Modifications to the Existing Signaling Network

#### 5.1.1. Engineering the Snf3p/Rgt2p Pathway

The d-xylose engineering attempts related to the Snf3p/Rgt2p pathway have primarily focused on making alterations to the hexose transporters. Several sugar transporter variants with single amino acid substitutions have been found that improve the affinity for d-xylose and its transport rate, including Hxt7p^F79S^, Hxt11p^N376T^, Hxt36^N367A^, and Gal2p^N376F^ [255,284,285,286]. Many of the hot spots found in these studies are located in highly conserved regions shared with the Snf3p and Rgt2p sensors, and these substitutions could therefore be relevant for engineering of the two sugar sensing membrane proteins.

Hxt1p/5p/36p have been engineered for increased longevity in the cell by removing ubiquitination sites via amino acid substitutions and thereby inhibiting the protein degradation signal. In particular, the Hxt36p^K12,35,56R^ mutant transporter resulted in improved d-xylose consumption rate [287]. While this example does not involve the Snf3p/Rgt2p pathway directly, it is an example of how proteins can be engineered to be less susceptible to signaling events that result in post-translational modifications such as ubiquitination and phosphorylation. More recently, Wu and colleagues deleted *RGT1* encoding the transcriptional repressor of *HXT* genes to simulate a d-glucose signal and derepress the expression of hexose transporters on d-xylose. The *rgt1Δ* strain achieved 23–24% higher d-xylose consumption rate both on d-xylose alone and during the d-xylose phase of mixed sugar cultivation [223], highlighting the importance of this target for yeast strain engineering.

#### 5.1.2. Engineering the SNF1/Mig1p Pathway

So far, Hxk2p has been the major target of SNF1/Mig1p pathway engineering. As was discussed in Section 4.1.2, interaction with D-xylose leads to irreversible autophosphorylation of Hxk2p and inactivation of the protein. Consequently, attempts have been made to create d-xylose resistant Hxk2p variants [36] while taking into account that a substitution of Ser158 is undesired as it results in decreased Hxk2p catalytical activity [130]. The best candidate from a screening of a Hxk2p amino acid substitution library, Hxk2p^F159Y^, had 64% higher activity when cultivated on a mixture of d-glucose and d-xylose compared to the wild-type variant, but only marginally improve d-xylose utilization [36]. This suggests that alteration of additional signals than just that of Hxk2p might be needed for improved d-xylose signaling via the SNF1/Mig1p pathway.

Efforts have also been made to constitutively localize Hxk2p to the nucleus: one such variant, Hxk2p^S15A^, resulted in improved growth, d-xylose consumption, and ethanol formation in an XI strain when grown on d-xylose. Transcriptome analysis further revealed that *XKS1* (encoding xylulokinase, Figure 1) was upregulated while genes related to respiration and glycerol production were downregulated in the Hxk2p^S15A^ strain [288]. Further deletion of *MIG1* was found to mitigate d-xylose utilization improvements, suggesting an interplay between these two SNF1/Mig1p pathway elements during d-xylose catabolism [288].

#### 5.1.3. Engineering the cAMP/PKA Pathway

The cAMP/PKA pathway has been a target of several engineering attempts since, in addition to sugar sensing, it regulates several growth and stress responses such as thermotolerance and inhibitor tolerance [289,290]. An evolutionary study that evolved XI strains for improved d-xylose utilization found loss-of-function mutations in the gene encoding the Ras1p/2p regulator Ira2p [249]. The improved d-xylose consumption rate phenotype was reproduced by deletion of *IRA2,* confirming the role of cAMP/PKA signaling on d-xylose utilization [249]. Introduction of the *IRA2* deletion in XR/XDH strains also resulted in improved d-xylose consumption rates and a double-deletion of *IRA2* and *ISU1* (another target identified in [249]) resulted in three times higher d-xylose consumption and ethanol production rates than in the background strain under anaerobic conditions [291]. *IRA2* deletion or inactivation leads to constitutive PKA activation, which deregulates the cAMP/PKA pathway [151,155,292,293]; indeed, the low fluorescence signal on D-xylose was changed to a high fluorescence signal, like the one observed at high D-glucose levels, in the Snf3p/Rgt2p and cAMP/PKA controlled biosensors when *IRA2* was deleted [291]. Comparable results were obtained when deleting both cAMP phosphodiesterase genes *PDE1/2* or using the Gpa2p^G132V^ mutant that leads to constitutive activation of the pathway but not when using the RAS2^G19V^ allele, which is expected to give high levels of cAMP and PKA activity, or when keeping one allele of the *PDE1/2* genes active [223]. In another study, Myers and co-workers found that the deletion of the negative PKA regulator-encoding gene *BCY1* led to remarkably high ethanol yield on d-xylose; however, this was accompanied by cell growth arrest under anaerobic conditions and poor aerobic growth on d-glucose [235]. In summary, the current engineering studies on the cAMP/PKA pathway have demonstrated that perturbations in the cAMP-PKA pathway via, for instance, *IRA2, PDE1/2* or *BCY1* deletions, can result in beneficial effect on d-xylose utilization rate or yield. However, complete deregulation of the pathway cannot be considered because the changes also lead to reduced stress tolerance and lower biomass formation [291,294,295,296]. The biosensor studies with *IRA2* also showed that these specific deletions only affect the cAMP/PKA pathway and not the global sugar signaling [291]. In the Bcy1p example disclosed above, it was also found that further perturbations in the *BCY1* gene sequence through gene fusion led to different effects on PKA than the complete deletion of the *BCY1* gene, with recovery of growth on d-glucose and high d-xylose fermentability [235]. Therefore, fine-tuning of PKA activation is essential to reach efficient d-xylose utilization without severe impact on growth and other industrially relevant properties.

### 5.2. Synthetic d-Xylose Signaling Networks

As a parallel strategy to modifying the endogenous signaling pathways, an increasing number of studies have used synthetic biology approaches to construct artificial regulatory and signaling circuits that render microbes sensitive to non-native substrates [297,298,299,300]. Synthetic signaling networks are defined as either: (i) a cascade of novel signaling events, or (ii) a set of exogenous or engineered TFs with new specificities and signals. Two different strategies for building synthetic d-xylose signal circuits in *S. cerevisiae* have so far been attempted: the first uses the bacterial transcription factor XylR [301,302,303] and the second uses a modified version of the native *S. cerevisiae GAL* regulon [259].

#### 5.2.1. XylR-Based Signaling Circuits

**Figure 7:**
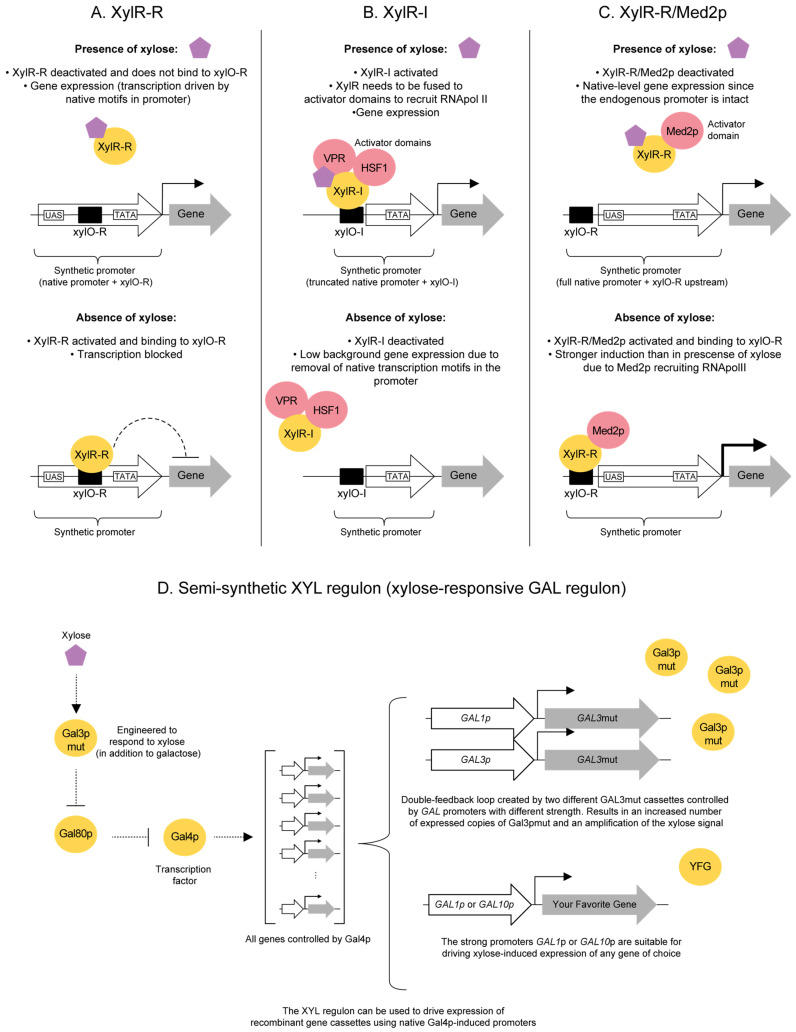
Schematic overview of strategies for synthetic d-xylose signaling circuits currently implemented in *S. cerevisiae.* (**A**) The repression-type XylR-R is used to block gene expression in the absence of d-xylose by binding to its operator *xylO-R*, which induces expression in the presence of d-xylose. Note that variations of the position of *xylO-R* in relation to the native elements can be used to tune the circuit strength [302]. (**B**) The yeast XylR-I strategy uses an *E. coli* activator-type XylR-I fused to activator domains. HSF1 is a mammalian heat shock factor 1 transactivation domain [308] and VPR is mammalian VP64-p65-Rta [309]. XylR-I is activated in the presence of d-xylose and binds to the *xylO-I* operator in the synthetic promoter and the activator domains recruit RNA polymerase II that initiates transcription. In the absence of d-xylose, XylR-I is inactivated and does not drive transcription. (**C**) The XylR-R/Med2p strategy combines elements from XylR-R and XylR-I by using a XylR-R fused to the Med2p activator domain. By positioning the *xylO-R* site upstream of the native promoter elements in the synthetic promoter, the circuit will be activated and drive gene expression in the absence of d-xylose. Presence of d-xylose will lead to XylR-R/Med2p deactivation and native levels of gene expression driven by the native operators of the promoter will occur. (**D**) The semi-synthetic *XYL* regulon utilizes a signaling protein (Gal3p) in the d-galactose regulon to respond to d-xylose (Gal3pmut). The native signaling in the regulon is kept intact but will now respond to d-xylose in addition to d-galactose. Solid arrows with arrowheads: induction; solid arrows with hammerheads: repression; dashed arrow with arrowhead: gene expression; orange pentagon: d-xylose. UAS: upstream activating sequence; TATA: TATA-box cis-regulatory element; YFG: Your Favorite Gene. Adapted from [259,302,303,306].

As discussed above in Section 4.2, the XylR sensor functions as a transcriptional repressor (XylR-R) in several d-xylose-utilizing bacteria (e.g., *C. crescentus* and *B. subtilis* [278,281]) (Figure 7A), and as a transcriptional inducer (XylR-I) in *E. coli* [275,276] (Figure 7B). Both types of XylRs have been successfully used to build small d-xylose-dependent regulation circuits in *S. cerevisiae,* based on binding of proteins to genomic motifs to achieve blocking or recruiting of RNA polymerase II (principles similar to that of RNA interference/CRISPR interference and RNA activation/CRISPR activation). XylR-R achieves interference by binding to DNA in the promoter regions of target genes and sterically blocking transcription. With XylR-I, the activation strategy relied on fusing activator domains capable of recruiting RNA polymerases to the DNA-binding site (Figure 7B) [304]. Both systems required the construction of tailor-made hybrid promoters by introducing XylR-binding motifs (called operators, or *xylO*) in native *S. cerevisiae* promoters.

The first XylR type, the gene repressing XylR-R (Figure 7A), was adapted for *S. cerevisiae* in 2015 by two independent groups [301,302] and several studies have since demonstrated the versatility of the system. The induction and repression responses could be varied by using XylR-Rs originating from different species [301,302], *xylO* sequences from different XylR-R hosts (*xylO-R*) as well as degenerated *xylO-R* sites [302]. The positioning of the motifs within the hybrid promoter could also be used to modulate the signal response. The highest induction ratio, as assayed with GFP, was found when the XylR-R DNA binding motif (*xylO-R*) was positioned directly upstream of the TATA-box [302]. This finding was further corroborated by later studies that expanded the available XylR-R hybrid promoters by using new yeast promoters as the basis for the synthetic promoter [305,306]. Addition of up to four tandem *xylO-R* motifs resulted in decreased expression of the XylR-controlled genes [306], possibly due to spatial limitations around the repeating *xylO-R* site. The choice of terminator also greatly affected the strength of the d-xylose-dependent induction assayed by GFP) as well as the level of background expression in the absence of d-xylose [307].

The second type of XylR, the gene expression-inducing XylR-I, was recently implemented in *Yarrowia lipolytica* and *S. cerevisiae* [303]. Whereas the XylR-R strategy relies on the XylR-R binding to the *xylO* motif and blocking transcription, expression of XylR-I in eukaryotes requires a fusion of the XylR protein to an activator domain capable of recruiting the endogenous RNA polymerase II (Figure 7B). A synthetic promoter with a *xylO-I* site, recognized by XylR-I, added upstream of the native *TEF1* promoter was used to drive GFP expression [303]. Mutant XylR-Is with higher affinity to their DNA-binding motif and strong induction response have also been identified in *E. coli* [277,310] and shown to have an alleviating effect on CCR [277], but these mutations remain to be tested in *S. cerevisiae*.

A third approach combines the XylR-R and the XylR-I strategies with the ambition to create a regulatory circuit that induces expression of XylR-controlled genes upon low levels of d-xylose [306]. Specifically, the authors fused the *St. xylosus* XylR-R [302] to the activation domain of the RNA polymerase II mediator complex subunit Med2p and used a hybrid promoter (*LEU2*p*-xylO-R*) to allow the XylR-R/Med2p complex to regulate expression of GFP (Figure 7C). During high d-xylose levels, the XylR-R/Med2p complex was repressed by d-xylose and only basal levels of GFP were observed since the hybrid promoter was engineered to lose its native activity. As d-xylose levels decreased, activated XylR-R/Med2p bound to the *xylO-R* site of the hybrid promoter which led to the recruitment of RNA polymerase II by Med2p and induced expression of GFP (Figure 7C) [306].

Despite their validation with GFP, no XylR circuits to date have been used to drive expression of d-xylose utilization pathways in *S. cerevisiae*. XylR has, however, been used to screen a mutant library of hexose transporters to find variants with improved d-xylose transport activity, since stronger induction of the circuit indicates increased uptake of d-xylose inside the cell where it bound to and deactivated XylR-R [302]. XylRs were also used to drive expression of TFs that affect the pentose phosphate pathway, but no significant difference in d-xylose utilization was observed [311]. Nevertheless, the principle of driving endogenous TFs by exogenous xylose-dependent sensors is a useful addition to the signaling engineering toolbox.

#### 5.2.2. GAL-Based Signaling Circuits

In addition to the XylR circuits, one study has engineered the *S. cerevisiae GAL* regulon to respond to d-xylose while retaining control over the expression of its native targets [259]. To reach this goal, Gopinarayanan and Nair used a biosensor approach to screen a library of Gal3p mutants for protein variants with increased sensing to d-xylose on top of the native d-galactose-binding [259]. By exchanging the native *GAL3* gene with the most responsive d-xylose-responsive mutant (*GAL3mut*), the authors were able to induce the native *GAL* regulon gene targets in the presence of d-xylose; the circuit was named the semi-synthetic *XYL* regulon (Figure 7D) [259]. Unlike the *S. cerevisiae* XylR-circuits discussed above, the *XYL* regulon was used to drive expression of a d-xylose utilization pathway. Using the xylose-responsive *GAL3mut*, the standard *S. cerevisiae GAL* expression system (galactose inducible *GAL1* and *GAL10* promoters) was used to express the genes of a d-xylose isomerase pathway (*XYLA*, *XKS1*, *TAL1*) and a d-xylose sensitive transporter (*GAL2-2.1*) by induction with d-xylose [259]. When compared to a control strain where the same genes were overexpressed by the constitutive *TEF1* and *TPI1* promoters, the growth rate on d-xylose was twice as fast for the double-feedback *XYL* regulon strain and d-xylose was consumed faster and to a higher degree than in the control strain [259].

## 6. Outlook

There are more and more indications that achieving well-performing microbial cell factories engineered to utilize non-native substrates requires not only functional expression of the heterologous metabolic pathway, but also engineering of the sensing and signaling networks. The major challenge of engineering non-native sensing is, however, that it requires an advanced understanding of the signaling of the native metabolites before any non-native signals can be understood. Based on the current status of the field reviewed above, three synergistic future directions for the research on d-xylose sensing in *S. cerevisiae* emerge: (i) increased efforts to elucidate the effects on d-xylose on the native signaling pathways and their subsequent engineering; (ii) development of synthetic signaling pathways that can operate orthogonally to the native systems; and (iii) computational modeling of signaling networks.

### 6.1. Towards Increased Understanding of d-Xylose Sensing

The research on the non-optimal d-xylose utilization in *S. cerevisiae* has reached a point where several hypotheses regarding metabolic issues have been addressed and to some extent resolved. Examples include the expression of various catabolic pathways from different hosts, the balancing of redox equivalents, the adjustments to the native pathways including the pentose phosphate pathway, the release of inhibition by xylitol and the expression of D-xylose transporters. As a consequence, the signaling and regulatory effects imposed by the d-xylose molecule on the cell increasingly appears as the final frontier that needs to be explored to solve this engineering challenge. This calls for more studies on the effect of d-xylose on the signaling networks of both wild-type and engineered *S. cerevisiae.* Excellent first steps have been taken (as reviewed in Section 4.1), but a substantial research effort likely remains before levels of understanding sufficient to drive systematic engineering of the native signaling networks to respond to d-xylose are reached. To achieve this, development of new methodologies to improve and speed up detection of signaling events will also be needed. Omics methods such as transcriptomics and phosphoproteomics are valuable tools for detection of signaling-induced transcription and signal transduction events, respectively. Nevertheless, they can quickly become technically and logistically challenging as multi-timepoint omics is still very costly, need many replicates and require large computational power for data analysis [312]. Methods that can increase the temporal resolution of signaling effects by allowing for frequent sampling during a cultivation will bring valuable knowledge of the d-xylose signaling dynamics. To this end, our group has developed a set of biosensors that measure the transcript level effects of signaling via GFP expression and flow cytometry [222,291]. However, while these biosensors measure the outcome of the full signal cascades, additional rapid methods for determining signal transduction events upstream in the networks would be highly useful to increase the molecular understanding of the effect of d-xylose on these pathways.

The four hypothetical mechanisms for d-xylose sensing in *S. cerevisiae* proposed in Section 4.1.5 can function as a roadmap for future research directions: (i) the d-xylose molecule itself can be recognized by some of the signaling pathways; (ii) d-glucose specific sensors can respond non-specifically to d-xylose due to the structural similarity of d-xylose and d-glucose; (iii) the different levels of the shared glycolysis and gluconeogenesis metabolites formed by the catabolism of d-glucose or d-xylose can be sensed by the signaling pathways; and iv) the different redox and energy carrier levels created during cultivation on the different sugars can be sensed as a signal of cellular homeostasis or well-being. We believe that the complexity of a microbial cell calls for holistic views of the molecular events of the cellular system and that the interactions between signaling networks and metabolic pathways need to be considered together, and not as two isolated parts. With that in mind, increased investigation of hypotheses three and four will be imperative to reach improved d-xylose utilizing and sensing strains.

In a review on engineering the *S. cerevisiae* MAPK signaling pathways, Furukawa and Hohmann identified five different approaches for signaling network engineering: assembly of regulatory elements, forced protein compartmentalization, systematic pathway modifications, heterologous expression of signaling cascades and rewiring of signaling transduction [313]. Of these five, the first four have been applied to d-xylose signaling engineering, as has been discussed in Section 5, including forced nuclear localization of Hxk2p, deletions of signaling elements, XylR synthetic circuits and engineering of the GAL regulon to respond to d-xylose. Up to now, most attention has been given to target elements of the cAMP/PKA pathway, which has led to strains with improved d-xylose utilization, but also industrially undesirable side traits such as decreased stress tolerance and biomass formation (Section 5.1). The SNF1/Mig1p and Snf3p/Rgt2p pathways have been less engineered, but current strategies to force the activation of a d-glucose signal during d-xylose cultivation has led to improved d-xylose consumption rates by modification of Hxk2p and deletion of *RGT1*, respectively [223,288]. The available studies in the cAMP/PKA pathway have highlighted the importance of building strains with different combinations of targets as only certain combinations led to improved d-xylose utilization [249]. Likewise, strains that combine elements of the current signaling engineering findings are yet to be attempted such as building a strain with a xylose pathway, combinations of *ira2Δ*, *isu1Δ*, *rgt1Δ* and with the modified Hxk2p^S15A^. The strong d-glucose catabolite repression in *S. cerevisiae* and other yeasts is another issue that is closely related to d-xylose utilization, especially for mixed-sugar cultivations; some progress towards CCR alleviation has already been reported in *Kluyveromyces marxianus* and *S. cerevisiae* [236,314,315] and it is likely that further CCR engineering will be required to improve mixed-sugar fermentation.

One of the suggested signaling engineering strategies [313] remains to be attempted for d-xylose signaling in *S. cerevisiae*: signal rewiring. Changing the signal transduction flow within the pathways to yield new physiological responses has great potential and applicability on the *S. cerevisiae* d-xylose utilization challenge. However, it requires a significantly more advanced understanding of the native signaling networks and their response to the non-native sugar d-xylose than what is currently known. Likewise, heterologous expression of complete d-xylose sensing pathways from closely related organisms that naturally utilize d-xylose (e.g., *Sc.stipitis, Sp. passalidarum* and *Candida tropicalis* [316]) is an ambitious milestone. This expression requires an in-depth mechanistic understanding of the signaling networks, both in the strain containing the recombinant network, and in the host-strain that the original network was taken from. While it cannot be overstated what a considerable fundamental and applied research effort this will require, signaling pathway “transplantation” could be a future cutting-edge goal for the cell signaling, metabolic engineering and systems biology communities.

### 6.2. Future Directions for Synthetic d-Xylose Signaling Networks

The high degree of interconnectivity and cross-talk in native signaling networks complicate their engineering, thereby synthetic (non-native) signaling circuits can provide a higher degree of pathway orthogonality, i.e., the ability to function independently of and in parallel to the native signaling and metabolic pathways [317]. This is especially the case for exogenous signaling elements, which is clearly illustrated in the difference between the bacterial XylRs and the semi-synthetic *XYL* regulon. Whereas the bacterial XylRs theoretically only affects the synthetic hybrid promoters containing *xylO* motifs, the d-xylose-sensitized Gal3p can still affect the native *GAL* regulon. This results in the expression of the *GAL* regulon’s many targets in addition to the recombinant material, such as the d-xylose-utilization pathway. This was indeed confirmed by a differential expression analysis of the constitutive and *XYL* regulon-controlled xylose pathway strains in which many expression differences related to the native targets of the *GAL* regulon were also found on d-xylose [259]. While the extent of background or unintended expression of native genes by the recombinant XylRs remains to be investigated, XylR circuits have an inherently higher degree of orthogonality than the *XYL* regulon. This orthogonality will likely lead to a lower level of background expression and a lower metabolic burden caused by expression of unrelated and/or undesired genes, ultimately giving the metabolic engineer more control over the circuit. Using non-native genetic material in a synthetic signaling circuit is not guaranteed to achieve orthogonality. However, as has recently been shown in *E. coli*, orthogonal synthetic signaling is achievable if testing of potential cross-talk between the heterologous material with the native pathways is part of the design [318].

Another benefit of synthetic signaling is that it can be applied before an advanced understanding of the native signaling has been achieved. Taking regulatory elements that have been characterized in other species and combining them with endogenous genetic elements can result in novel signaling effects [313]. However, synthetic d-xylose signaling in *S. cerevisiae* is still at its infancy with a level of complexity many factors lower than the multi-element cascades of the native sugar signaling networks. The XylR circuits, for instance, which could be considered the only fully synthetic d-xylose signaling circuit in *S. cerevisiae* to date since all its regulatory elements (XylR and *xylO*) are of exogenous origin, only cover the final step of a gene regulating signaling cascade:TF-controlled gene expression [301,302,303,305,306]. However, since the different XylR strategies remain to be applied to drive d-xylose utilization, it is currently not known if a circuit containing a single signaling element (the XylR) is enough to improve d-xylose utilization in *S. cerevisiae,* which is the case in e.g., *E. coli* [275]; more complex circuits with several signaling elements or loops might instead be required to reach a sufficient regulation. In *B. subtilis*, for instance, a multi-step XylR-based circuit using two regulators that each control a promoter has been successfully implemented [319]. Now that several regulatory elements from four different synthetic d-xylose signaling circuits (Figure 7) have been demonstrated, there should be enough pieces available to build higher complexity circuits also in *S. cerevisiae.* Thus, the next big milestone for these synthetic d-xylose signaling circuits would not be the identification of additional engineering strategies, but the combination of the existing ones into networks that closer resemble the regulatory complexity of native signaling networks.

### 6.3. Computational Modeling of Sugar Signaling?

Mathematical modeling of the cellular metabolism is extensively used to drive strain design in metabolic engineering and systems biology and can be used to simulate and predict systemic effects of changes to the metabolic pathways such as adding new reactions and deleting existing ones [320]. In silico flux analyses of genome-scale reconstructions of the *S. cerevisiae* metabolism have been used to identify potential metabolic bottlenecks. For instance, in recombinant d-xylose utilization, such analyses have highlighted the effect of the inherent NAD(P)H imbalance in the first generations of the XR/XDH pathway [311,321,322,323]. However, these models have historically mainly taken metabolic pathways into account. If we consider all the examples from this review of the impact of signaling on recombinant d-xylose utilization, it becomes clear that a mathematical model that can combine metabolism, signaling and gene regulation would be needed to better simulate systemic effects of the non-native sugar d-xylose.

So called hybrid models that integrate reconstructions of both the metabolic and the signaling networks and are able to take metabolic flux and signal transduction into account have indeed been in the works for some time and are increasing in complexity [324,325,326,327]. The challenge with implementing these models is that metabolic and signaling networks have intrinsically different mechanisms of action and require distinct modeling methods [56,320]. Metabolism is a mass flow controlled by chemical reactions where rates, kinetics, concentrations and thermodynamics are often modeled with constraint-based stoichiometric models [320]. Signaling networks, on the other hand, consist of a signal flow operating by phosphorylation, activation and repression, and are often represented by Boolean logic statements (e.g., TRUE, FALSE, AND, OR, NOT; [56,328]). A few *S. cerevisiae* signaling reconstructions have been made, including the Snf3p/Rgt2p pathway [329], the SNF1/Mig1p pathway [328,329], the cAMP/PKA pathway [189] and the osmotolerance HOG pathway [330]. The ubiquitous cross-talk between signaling networks does however complicate modeling, and to make a reconstruction of a specific signaling pathway, a number of cross-talking pathways would have to be included in order to achieve a good level of agreement with experimental data. The SNF1/Mig1p-Snf3p/Rgt2p pathway cross-talk was one of the first to be reconstructed, using Boolean logic [329], and later models that include cross-talk of three main sugar signaling pathways, SNF1/Mig1p, Snf3p/Rgt2p and cAMP/PKA, have been made [331].

Optimally the *S. cerevisiae* sugar signaling-metabolism hybrid models could be expanded to include the reactions of the recombinant d-xylose pathways and their interactions with the native signaling pathways The resulting *S. cerevisiae* d-xylose hybrid model could then become a highly useful systems biology tool to identify targets for signaling engineering.

## 7. Conclusions

To achieve industrially and societally relevant bioprocesses, microbial cell factories often need to be engineered with expanded substrate ranges. However, once functional expression of novel catabolic pathways has been achieved, substantial molecular optimization is typically required to reach economically feasible yields, titers and productivities. To be able to achieve these levels of optimization, we foresee that engineering of the sensing of non-native substrates shall be an essential component of the metabolic engineering and systems biology strategies. d-Xylose sensing by engineered *S. cerevisiae* has a good potential to become a golden standard in the field of non-natural substrate sensing and signaling because substantial metabolic engineering achievements are already in place owing to many decades of research progress; also, the native d-glucose sensing is already a well-studied topic in this yeast. While a lot remains to be understood of the signaling responses to different sugars before rational signaling engineering can be attempted at a larger scale, the first steps towards that end have already been taken as has been illustrated by this review.

## Figures and Tables

**Table 1:** Batch cultivation performances for some of the best reported *S. cerevisiae* strains engineered for d-xylose utilization, by rational engineering and laboratory evolution. Under anaerobic conditions, the best reported strains (XR/XDH and XI strategies) reach ~80% of the maximum ethanol yield (0.51 g EtOH g^−1^ d-xylose). Productivity, however, remains low compared to values for d-glucose where production rate of ethanol and consumption rate of d-glucose can reach 1.2–2.5 g EtOH g^−1^ CDW h^−1^ and 3 g d-glucose g^−1^ CDW h^−1^, respectively [40]. * A xylonate formation rate from d-xylose of 0.11 g L^−1^ h^−1^ (1:1 stoichiometry) was reported but no data on cell dry weight [16]. Note that the table does not reflect on the capacity of these strains for d-glucose/d-xylose co-utilization. XI: xylose isomerase; XR/XDH: xylose reductase/xylitol dehydrogenase; CDW: cell dry weight; N/A: not applicable.

Strain	d-Xylose Pathway (and Subsequent Evolution)	Oxygenation	Maximum Specific Growth Rate (µ_max_) on d-Xylose (h^−1^)	d-Xylose Consumption Rate (g d-Xylose g ^−1^ CDW h ^−1^)	Yield (g EtOH g^−1^ d-Xylose)	Specific Ethanol Production Rate (g EtOH g^−1^ CDW h^−1^)	Reference(s)
Anaerobic d-xylose assimilation via the pentose phosphate pathway							
RWB 217	XI (non-evolved)	Anaerobic	0.09	1.06	0.43	0.46	[41,42]
H131-A3-AL^CS^	XI (evolved)	Anaerobic	0.20	1.87	0.41	0.77	[43]
TMB 3504	XR-XDH (non-evolved)	Anaerobic	0.11	0.76	0.40	0.33	[44]
SR8	XR-XDH (evolved)	Anaerobic	0.09	0.87	0.31	0.28	[45]
Aerobic d-xylose oxidation							
TMB4590	Weimberg pathway	Aerobic	0.08	0.16	N/A		[17]
H4099	Dahms pathway	Aerobic	No growth	Specific rate not reported *	N/A		[16]

**Table 2:** Main metabolic and physiological effects of PKA activation. PKA is activated via sensing of extracellular d-glucose and glycolytic activity. Signaling pathway cross-talk is not covered by this table. Adapted from [78].

**Targets induced/activated by active PKA**	Ribosome biogenesis and ribosomal protein genes	[131,132]
	*BAT1* gene (Tpk1p regulation): gene involved in exit from stationary phase, iron homeostasis and mitochondrial DNA stability	[133]
	Pseudohyphal growth (Tpk2p regulation)	[133]
	Genes involved in trehalose degradation and water homeostasis (Tpk2p regulation)	[133]
	Growth and increase of biomass	[131,134]
	Low-affinity hexose transporters via Rgt1p phosphorylation (e.g., *HXT1*)	[106]
	Glycolytic enzyme, e.g., by phosphorylation of Pfk26p and Nth1p, and transcriptional upregulation of Pdc1p	[67,135,136,137]
	Protein phosphatases (PP2A and PP1), specifically dephosphorylating serine/threonine amino acids	[138]
**Targets repressed/inactivated by active PKA**	Enzymes involved in gluconeogenesis (fructose 1,6-bisphosphatase, isocitrate lyase)	[139,140,141]
	Stress-responsive genes (e.g., *MSN2/4*)	[142]
	Glycogen accumulation	[142]
	Rim15p (a protein kinase involved in adaptation process to enter in the stationary phase)	[143]
	Genes involved in iron uptake (Tpk2p regulation)	[133]
	Heat-shock genes (e.g., *HSP12, HSP26)* by inactivating the transcriptional activator Hsf1	[144]
	Transcription of genes involved in trehalose synthesis and accumulation (*TPS1/2);* Trehalose-6-phosphate synthase activity through phosphorylation of one of the regulatory subunits (Tps3p)	[145,146]
	*SUC2* (encoding invertase)	[147]
	Sak1p and SNF1 proteins	[148]

**Table 3:** Genes found to be upregulated or downregulated in xylose reductase/xylitol dehydrogenase (XR/XDH)strains in the presence of d-xylose. * Note that *MTH1* and *HXT2* were found to be upregulated and downregulated in different studies. Adapted from [78].

	Genes Related to:	References
Upregulation	Gluconeogenesis	[35,37,214,217]
	Genes related to the oxidative pentose phosphate pathway	[214,217]
	TCA and glyoxylate cycle	[35,37,38,217]
	Respiration	[35,37,38,217]
	Acetaldehyde and acetyl-CoA metabolism	[35,217]
	Genes typically expressed on non-fermentable carbon sources: *SUC2, HXK1*, *HXT5, HXT13,* maltose metabolism genes	[35,38,217]
	Sugar signaling: *MTH1* *, *ADR1, CAT8, RGT1*	[35,38,217]
	High-affinity d-glucose transporters (e.g., HXT2 *, *HXT6* and *HXT7*)	[35,38]
Downregulation	Glycolysis	[35]
	Low-affinity d-glucose transporters (e.g., *HXT1* and *HXT3*)	[38,217]
	Sulfur metabolism	[217]
	Heme biosynthesis from uroporphyrinogen	[217]
	Tryptophan degradation	[217]
	Sugar signaling: MTH1 *, *STD1, MIG1, HXK2*	[35,217]

## Data Availability

Not applicable.

## Abbreviations

cAMP: cyclic AMP; CCR; carbon catabolite repression; GFP; green fluorescent protein; HOG; high osmolarity/glycerol; MAPK; mitogen-activated protein kinase; PKA; protein kinase A; TCA; tricarboxylic acid (cycle); TOR; target of rapamycin; TF; transcription factor; UAS; upstream activating sequence.
